# Myosin-independent stiffness sensing by fibroblasts is regulated by the viscoelasticity of flowing actin

**DOI:** 10.1038/s43246-024-00444-0

**Published:** 2024-01-15

**Authors:** Nikhil Mittal, Etienne B. Michels, Andrew E. Massey, Yunxiu Qiu, Shaina P. Royer-Weeden, Bryan R. Smith, Alexander X. Cartagena-Rivera, Sangyoon J. Han

**Affiliations:** 1 Department of Biomedical Engineering, Michigan Technological University, Houghton, MI, USA.; 2 Health Research Institute, Michigan Technological University, Houghton, MI, USA.; 3 Section on Mechanobiology, National Institute of Biomedical Imaging and Bioengineering, National Institutes of Health, Bethesda, MD, USA.; 4 Department of Biomedical Engineering, Michigan State University, Lansing, MI, USA.; 5 Department of Mechanical Engineering and Engineering Mechanics, Michigan Technological University, Houghton, MI, USA.

## Abstract

The stiffness of the extracellular matrix induces differential tension within integrin-based adhesions, triggering differential mechanoresponses. However, it has been unclear if the stiffness-dependent differential tension is induced solely by myosin activity. Here, we report that in the absence of myosin contractility, 3T3 fibroblasts still transmit stiffness-dependent differential levels of traction. This myosin-independent differential traction is regulated by polymerizing actin assisted by actin nucleators Arp2/3 and formin where formin has a stronger contribution than Arp2/3 to both traction and actin flow. Intriguingly, despite only slight changes in F-actin flow speed observed in cells with the combined inhibition of Arp2/3 and myosin compared to cells with sole myosin inhibition, they show a 4-times reduction in traction than cells with myosin-only inhibition. Our analyses indicate that traditional models based on rigid F-actin are inadequate for capturing such dramatic force reduction with similar actin flow. Instead, incorporating the F-actin network’s viscoelastic properties is crucial. Our new model including the F-actin viscoelasticity reveals that Arp2/3 and formin enhance stiffness sensitivity by mechanically reinforcing the F-actin network, thereby facilitating more effective transmission of flow-induced forces. This model is validated by cell stiffness measurement with atomic force microscopy and experimental observation of model-predicted stiffness-dependent actin flow fluctuation.

Mechanical stiffness of the extracellular matrix (ECM) regulates many cellular functions such as spreading^1,2^, differentiation^3^, proliferation^4,5^ and migration^6^. In response to the ECM stiffness, different amount of mechanical tension is applied through integrin-based focal adhesions (FAs), which trigger the different levels of the conformational opening of mechanosensitive proteins such as talin and vinculin^7-13^. Indeed, multiple studies have found that cell-ECM adhesion transmits increasing traction in response to an increasing ECM stiffness^14-16^, which we term ‘stiffness-dependent differential traction’. To provide mechanistic understanding about the stiffness-dependent differential traction transmission, nonmuscle myosin-II contractility has been suggested as the main force-generator in many conceptual/multiphysics models^17-22^ including the molecular clutch model^17-22^. In addition to its role as a force generator, however, myosin II is also a major effector in response to the signals generated from mechanotransduction. Differential tension triggers the integrin-signaling such as RhoA-ROCK pathways^23,24^ or Ca^2+^- MLC kinase pathway^25^, which all activates myosin contractility by phosphorylating myosin light chain^26^. Additionally, the tension in F-actin, which would be proportional to the tension in FAs, promotes myosin’s localization to F-actin itself^27^ and prevent dissociation from F-actin^28^. Myosin activation and the force from it promotes cytoskeletal reinforcement and maturation of nascent adhesions to stronger FAs by recruiting other signaling and structural proteins^29^. Thus, myosin might be further activated in response to signaling from differential tension, which is again from myosin-based F-actin flow and clutching. Altogether, the dual roles of myosin as both an input and an output of stiffness sensing, complicates the understanding of the true source of the stiffness-dependent differential traction transmission.

As an alternative power source, actin assembly at the barbed end of F-actin can induce retrograde flow by pushing the membrane and being pushed by the membrane, which transmits the traction via cell-ECM adhesions^30^. Interestingly, myosin-II-inhibited cells are able to transmit reduced but significant traction force^31,32^. Additionally, indirect evidence shows that myosin-II inhibited embryonic fibroblasts exert traction seemingly increasing with the substrate stiffness^33^. In the absence of myosin-II activity, adherent cells also have been shown to exhibit increasing level of cell spreading in response to increasing ECM stiffness with denser adhesion assembly^34,35^. These findings suggest a possibility where the differential tension might be developed in cell-ECM adhesions in response to the ECM stiffness solely by actin polymerization-based retrograde flow and clutch between the flow and the adhesions. The two mediators for actin polymerization are actin-related-protein 2/3 (Arp2/3) complex and formin homology protein^36,37^. The Arp2/3 complex mediates formation of branched actin filaments just beneath the cell membrane^38^. Formin promotes linear actin assembly by catalyzing actin polymerization just beneath the F-actin’s growing barbed end^39,40^. Both Arp2/3 and formin mediate the advance of the F-actin network in a spatiotemporally-coordinated fashion^41^. However, how they regulate the traction through cell-ECM adhesions have been unclear.

In this paper, we systematically investigate myosin-II and actin nucleators for their roles in differential force transmission in response to ECM stiffness. We present a newly-developed molecular clutch model which takes into account the role of polymerizing actin and its viscoelasticity in stiffness-dependent differential force transmission in a myosin-independent manner. We show that stiffness-dependent traction transmission is still present without myosin-II contractile activity, and it is governed by actin polymerization mediated by Arp 2/3 and formin. We show that the force sensitivity to the ECM stiffness gradually decreases as we inhibit functions of Arp2/3, formin and actin polymerization itself in addition to myosin-II inhibition. Using the new computational model, we demonstrate that actin nucleators participate in mechanosensing by modulating the F-actin’s viscoelasticity. We further provide evidence of the new model by measuring the F-actin network elasticity via atomic force microscopy and by showing the fluctuation of the actin flow that relies on the ECM stiffness.

## Results

### Stiffness-dependent differential force transmission is independent of myosin-II activity.

**Fig. 1:**
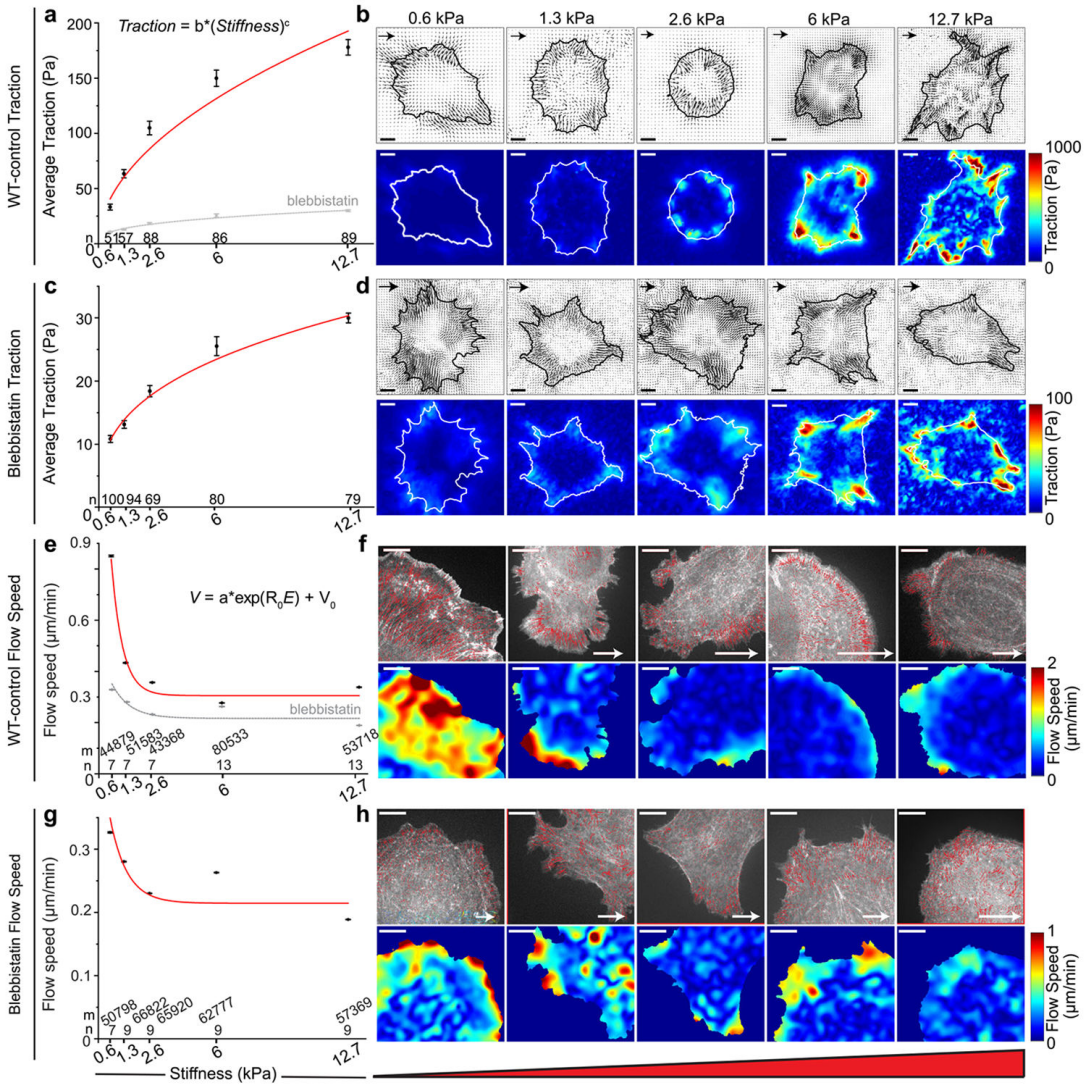
Stiffness-dependent differential traction is transmitted independently of myosin-II contractility in F-actin retrograde flow-dependent manner. **a** Average traction integrated over 1-μm-thick cell perimeter of WT NIH 3T3 fibroblasts as a function of different gel stiffness (red). Sample sizes, n, are denoted on top of each stiffness value. Markers with error bars: mean ± s.e.m. **b** Representative traction vector fields (top) and traction magnitude maps (bottom) of WT-control cells. Arrow scale: 150 Pa, 300 Pa, 500 Pa, 1500 Pa and 1500 Pa of traction for gel stiffness of 0.6 kPa, 1.3 kPa, 2.6 kPa, 6 kPa and 12.7 kPa, respectively. Scale bar: 10 μm. **c** Average traction integrated over cell perimeter of cells treated with 20 μM blebbistatin (BBS) as a function of a gel stiffness (grey dotted in **a** and red in **c**). **d** Representative traction vector fields (top) and traction maps (bottom) of BBS-treated cells. Arrow scale: 50 Pa, 75 Pa, 90 Pa, 100 Pa and 150 Pa of traction for gel stiffness of 0.6 kPa, 1.3 kPa, 2.6 kPa, 6 kPa and 12.7 kPa, respectively. Power-law curve fits (Traction = *b* * (Stiffness)^*c*^) were added in (a) and (c). See Supplementary Table 1 for fit parameters. **e** Average F-actin flow speed as a function of the gel stiffness of WT-control cells (red, n=7, 7, 7, 13, 13 cells for increasing stiffness, collected from m=44,879, 51,583, 43,368, 80,533, 53,718 windows). Markers with error bars: mean ± s.e.m., based on the number of cells where each cell has average speed averaged by all window samples in the cell. **f** Representative interpolated flow vectors (top) and speed maps (bottom) of SNAP-actin of WT 3T3 fibroblasts on a gel with increasing stiffness. Arrow scale: 5 μm/min of actin flow. **g** Average F-actin flow speed as a function of the gel stiffness of BBS-treated cells (grey dotted in (**e**) and red in (**g**), n=7, 9, 9, 9, 9 cells for increasing stiffness, m=50798, 66822, 65920, 62777, 57369 windows). A negative exponential function ($V=a*\text{exp}(R_{0}E)+V_{0}$) was used for flow speed vs stiffness plots in **e** and **g** (See Supplementary Table 2 for fit parameters). **h** Representative interpolated flow fields (top) and speed maps (bottom) of BBS-treated cells. Arrow scale: 3 μm min^−1^ of actin flow. Scale bar: 10 μm.

To confirm the stiffness-force relationship in wild-type (WT) cells^14-16^, we measured the traction of NIH 3T3 fibroblasts plated on a high-refractive-index silicone gel, coated with 40 nm-diameter fluorescent beads and fibronectin, for 4 hours with varying elastic moduli, e.g., 0.6 to 12.7 kPa. The bead images were analyzed for deformation and traction using correlation-based particle tracking velocimetry with re-tracking (cPTVR)^42^ and L_2_-regularized fast boundary element method (FastBEM)^43,44^ with an L-curve-based selection of an optimal regularization parameter, respectively^44,45^. The average traction over the cell periphery, i.e., 2 μm in width along the cell edge, where integrin adhesions are usually present, was found to increase with the gel stiffness (Fig. 1a). Specifically, a linear increase was observed up to 2.6 kPa, after which the average traction plateaued at the stiffer regime in 6 and 12.7 kPa (Fig. 1a), overall following a power-law relationship (adj. *R*^*2*^ = 0.99; Supplementary Table 1). This stiffness-dependent traction trend is consistent with data from previous studies^14-16,21^, and we term it as ‘stiffness-dependent differential traction’. As previously observed^14^, high tractions were mostly located at the cell periphery, which increased in response to the substrate stiffness (Fig. 1b).

To test whether the stiffness-dependent differential traction still exists in the absence of myosin-II activity, we treated the cells with 20 μM of blebbistatin (BBS), a myosin ATPase inhibitor^46,47^ for 1 hour after plating the cells for 3 hours, and measured the traction of the cells on gels with the same range of the gel stiffness. This BBS concentration has been shown to effectively suppress myosin-generated traction in fibroblasts^48,49^, while a higher dosage has been shown to cause a cytotoxic effect^50^ (see Materials and Methods, ‘Perturbations using small-molecular inhibitors’ section for further justification). The inhibition significantly reduced the magnitude of the traction overall, as expected (Fig. 1a, *a gray line*). The reduction was more substantial in the stiff regime (6-12.7 kPa, ~20 %) than in the intermediate (1.3-2.6 kPa, ~23 %) and softer regime (0.6 kPa, ~31 %) compared to the WT conditions (Supplementary Fig. 1). Interestingly, however, the myosin-II-inhibited cells still exhibited stiffness-dependent differential traction with a power-law trend (Fig. 1c) (adj. *R*^*2*^ = 0.98; Supplementary Table 1). The traction was distributed spatially similar to those shown by WT cells, i.e., inward and concentrated near cell edges (Fig. 1d), implying that myosin-inhibited traction is transmitted through integrin-based adhesions. Taken together, this result demonstrates that the stiffness-dependent differential traction is myosin-II-independent.

### F-actin retrograde flow speed decreases with increasing ECM stiffness in the presence and absence of myosin-II contractility.

F-actin retrograde flow is a major input to the traction transmission^32^. To evaluate how much myosin-II activity affects the actin flow as a function of ECM stiffness, we labeled F-actin in NIH 3T3 fibroblasts using SNAP-tag, visualized only a subset of them using a low SNAP substrate concentration^51^, and analyzed the F-actin time-lapse images of single cells with quantitative fluorescence speckle microscopy software (qFSM)^51,52^ for the actin flow field (Supplementary Movie 1). Imaging was performed 1 hour after plating to observe actin speckless to avoid well-developed stress fibers. As expected, WT cells showed an inverse relationship of the actin flow speed, V, with the stiffness, E, i.e., a decreased flow speed with increasing stiffness, followed by little change at high stiffness (Fig. 1e). A negative exponential function could well-represent this behavior (Fig. 1e, $V=aexp\left(R_{o}E\right)+V_{o}$ where a, $R_{o}$ and $V_{o}$ are fit constants). Most of the high flow velocity vectors were present at the leading edge of lamellipodia, e.g., ~1 μm width along the cell periphery (Fig. 1f, Supplementary Fig. 2), whereas there was a profound reduction in the flow speed at the cell-inner area further apart (>1 μm) from the cell edge (Supplementary Fig. 2a). Our observations of WT actin flow trends align with those previously documented in mouse embryonic fibroblasts and are coherently explained by the molecular clutch model, as demonstrated by^21^. Specifically, the trend we observed suggests an inverse-like relationship between actin flow speed and substrate stiffness. This implies that the observed stiffness-dependent differential traction is modulated by actin flow, reaffirming the model’s prediction that at higher substrate stiffness, actin flow becomes slower yet more effectively transmits traction forces due to increased coupling between actin and adhesion complexes.

In a similar manner to WT cells, cells treated with BBS exhibited a decrease in flow speed as the gel stiffness increased. This trend was observed until reaching 2.6 kPa, after which there was minimal change in flow speed (Fig. 1g, Supplementary Movie 2). The magnitudes of both the initial downward rate (coefficient A in the equation at Fig. 1g) and the exponential rate (coefficient $R_{o}$) decreased compared to those in WT (Supplementary Table 2). Despite this reduction, the negative exponential trend demonstrates that the stiffness-dependent differential traction is associated with the actin flow dynamics. As in WT (Fig. 1f), high flow speed regions were mostly at the 1 μm-width layer from the cell edge (Fig. 1h). Together, these results suggest that the traction existing in the absence of myosin-II activity is stiffness-dependent and also flow-dependent.

### Actin polymerization by Arp2/3 and formin contributes to stiffness-dependent differential traction.

Besides myosin-II contractility, actin polymerization can also generate the F-actin retrograde flow by propelling the cell plasma membrane and being pushed back by the membrane tension^53^. Inhibition of actin polymerization has been shown to result in a reduction in traction force in many cells types^54-56^, including fibroblasts^57^. However, the relative contribution of actin polymerization to traction force by fibroblasts and its potential dependency on the ECM stiffness have not been clearly understood. To identify whether and how much actin polymerization regulates stiffness-dependent, myosin-II-independent traction transmission, we treated cells (in addition to myosin-II- inhibition with BBS) with CK666 or SMIFH2, inhibitors for Arp2/3 or formin, the two main nucleators of F-actin^58^, or with Latrunculin-A (LatA), which inhibits actin polymerization and promotes actin depolymerization, and measured the traction of the cells 1 hour after treatment. While SMIFH2 can influence myosin-II at high concentration, e.g., >50μM^59^, the dose used in this study (20 μM) was low enough to avoid its effect on myosin-II contractility. Moreover, the experiments with SMIFH2 were performed in the presence of a separate myosin-II inhibitor BBS. Thus, the effect of SMIFH2 on myosin-II was considered minimal.

**Fig. 2:**
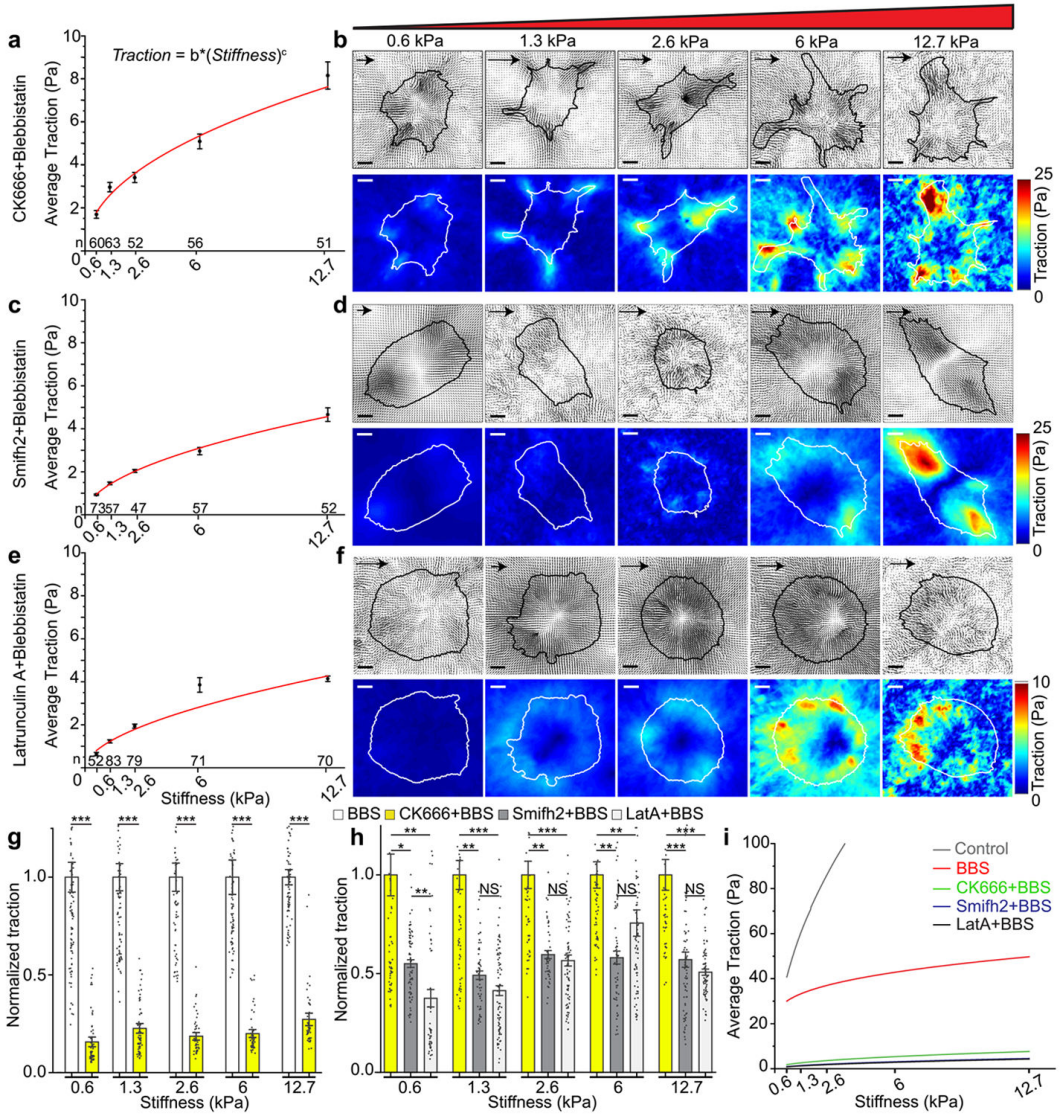
Myosin-independent, stiffness-dependent differential traction depends on actin polymerization mediated by Arp2/3 and formin. **a** Average traction integrated over cell perimeter of cells treated with 100 μM CK666 in addition to 20 μM BBS as a function of a gel stiffness. Sample sizes, i.e., the number of cells, n, are denoted on top of each stiffness value. Markers with error bars: mean ± s.e.m. **b** Representative traction vector fields (top) and traction magnitude maps (bottom) of CK666- and BBS-treated cells. Arrow scale: 10 Pa, 30 Pa, 40 Pa, 50 Pa and 75 Pa of traction for gel stiffness of 0.6 kPa, 1.3 kPa, 2.6 kPa, 6 kPa and 12.7 kPa, respectively. **c** Average traction of cells treated with 20 μM SMIFH2 in addition to 20 μM BBS as a function of a gel stiffness. **d** Representative traction vector fields (top) and traction magnitude maps (bottom) of SMIFH2- and BBS-treated cells. Arrow scale: 5 Pa, 15 Pa, 20 Pa, 30 Pa and 50 Pa of traction for gel stiffness of 0.6 kPa, 1.3 kPa, 2.6 kPa, 6 kPa and 12.7 kPa, respectively. **e** Average traction of cells treated with 1 μM Latrunculin-A (LatA) in addition to 20 μM BBS as a function of a gel stiffness. **f** Representative traction vector fields (top) and traction magnitude maps (bottom) of LatA and BBS-treated cells. Arrow scale: 3 Pa, 5 Pa, 7 Pa, 10 Pa and 25 Pa of traction for gel stiffness of 0.6 kPa, 1.3 kPa, 2.6 kPa, 6 kPa and 12.7 kPa, respectively. Power-law curve fits (Traction = *b* * (Stiffness)^*c*^) were added in (**a**), (**c**) and (**e**) (See Supplementary Table 1 for fit parameters). Scale bar: 10 μm. **g** Normalized average traction of BBS-treated cells (white) and CK666-BBS (yellow). Bar with error bars: mean ± s.e.m., *: *p* < 0.05, **: *p* < 1×10^−10^,***p < 1×10^−30^ by Mann-Whitney U test. **h** Normalized average traction of CK666-BBS-(yellow), SMIFH2-BBS-(dark grey) and LatA-BBS-treated cells (light grey). Error bars: s.e.m. *p < 0.05, **p < 1×10^−10^, ***p < 1×10^−20^ by Kruskal-Wallis ANOVA test with Dunn’s post-hoc analysis. **i** A plot of curve fits of average traction as a function of the stiffness for all conditions.

Cells subjected to combined treatment with CK666 and BBS demonstrated a significant reduction (approximately 70%) in traction across the range of stiffness compared to cells treated solely with BBS (Fig. 2a,g). This finding confirms the contribution of Arp2/3-mediated actin polymerization to myosin-II-independent traction transmission. However, even with this dual inhibition, the cells still exhibited a consistent increase in traction with increasing stiffness (Fig. 2a, b). When cells were treated with CK689, an inactive control for CK666, in addition to BBS, they displayed a stiffness-force trend similar to the BBS-only condition, with minimal reduction in traction (Supplementary Fig. 3). Furthermore, cells treated with SMIFH2 and BBS exhibited even lower traction compared to cells treated with BBS and CK666, showing a reduction of approximately 40% (Fig. 2c, h). Despite this, these cells still displayed stiffness-dependent differential traction (Fig. 2c, d). Most of the traction in both double-inhibited cells was distributed along the periphery of the individual cells, where major F-actin retrograde flow takes place^52,60^ (Fig. 2b, d), suggesting that the traction is transmitted via cell-ECM adhesions via the molecular clutch mechanism. Specifically, formin activity appeared to play a more crucial role than Arp2/3 in regulating myosin-II-independent stiffness-force sensitivity (Fig. 2h, *yellow vs. gray bars*). This formin’s stronger mechanosensitivity than Arp2/3 may stem from formin’s role in regulating filament length^61^ and length-dependent stiffening where longer filaments accommodate more flexible crosslinks, enhancing load resistance^62^ (See the 3^rd^ paragraph in Discussion).

In order to assess the extent of actin polymerization’s contribution to myosin-II-independent stiffness sensing, we disrupted actin polymerization by treating the cells with LatA in combination with BBS. LatA not only sequesters actin monomers (G-actin) but also accelerates F-actin depolymerization^63^. This combined inhibition resulted in a further reduction in average traction, slightly lower than that observed in cells with formin-myosin-II inhibition, except for one stiffness condition (6 kPa) (Fig. 2e, h). However, cells treated with LatA-BBS still displayed stiffness-dependent differential traction (Fig. 2e) with most of the force distribution concentrated along the cell periphery (Fig. 2f). These findings indicate that actin polymerization significantly contributes to stiffness-dependent force sensitivity (as summarized in Fig. 2i), but it is not the sole factor. Taken together, these traction data suggest that myosin-II-independent, stiffness-dependent differential traction relies on actin polymerization to a great extent, but the stiffness-dependence is minimally present due to additional mechanosensitive mechanisms beyond actin polymerization.

### Formin activity and actin polymerization are required for stiffness-dependent F-actin retrograde flow speed changes while Arp2/3 activity is partially necessary.

**Fig. 3:**
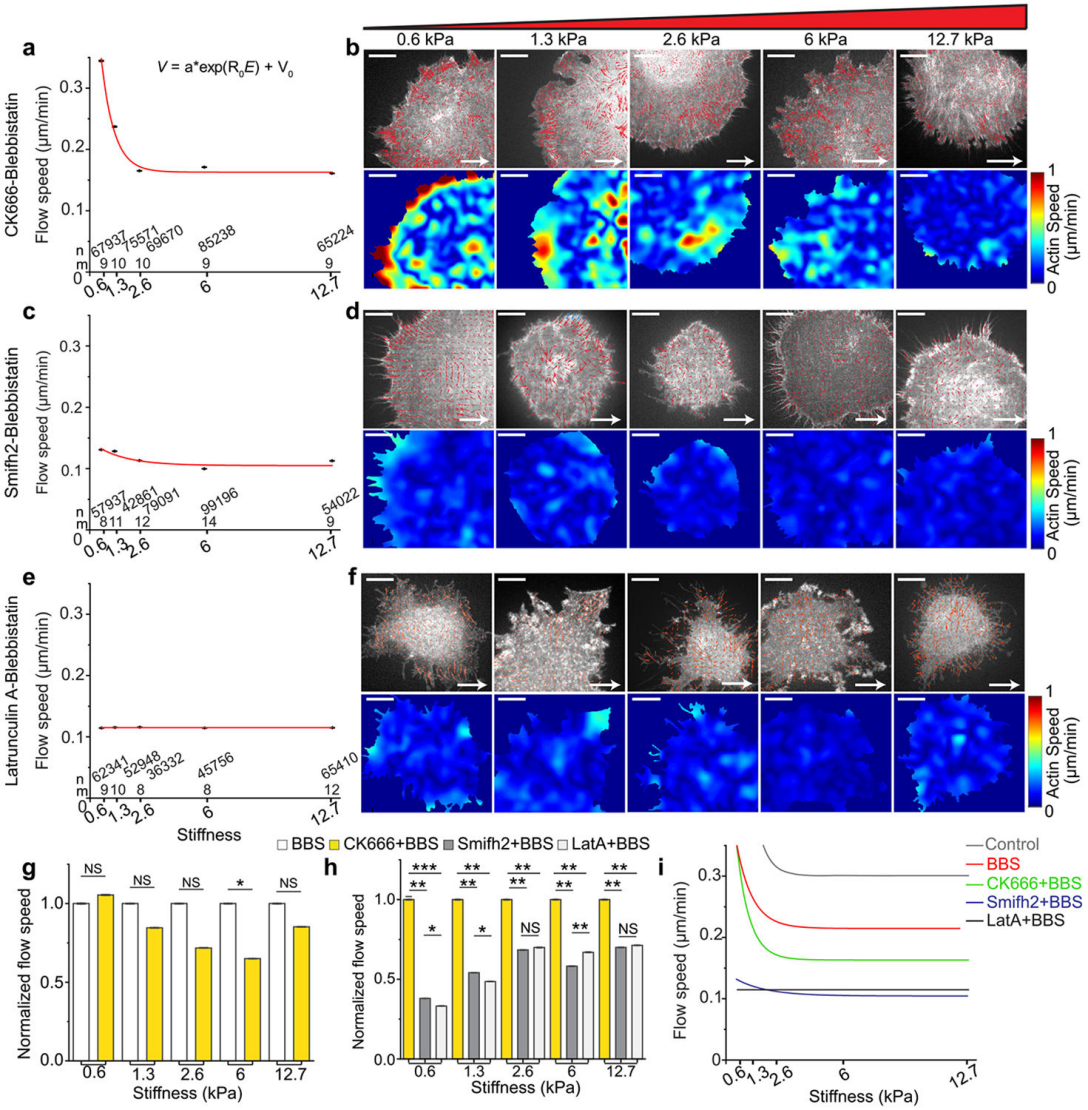
Myosin-independent F-actin retrograde flow is still stiffness-dependent in the absence of Arp2/3 but becomes negligible in the absence of formin and actin polymerization. **a** Average F-actin flow speed as a function of the gel stiffness of CK666-BBS treated cells (red, n=9, 10, 10, 9, 9 cells for increasing stiffness, collected from m=67937, 75571, 69670, 85238, 65224 windows). Markers with error bars: mean ± s.e.m. where the statistics was based on the number of cells. **b** Representative interpolated flow vectors (top) and speed maps (bottom) of SNAP-actin of CK666-BBS-treated fibroblasts on 0.6 kPa, 1.3 kPa, 2.6 kPa, 6 kPa and 12.7 kPa gel. Arrow scale: 3 μm min^−1^ of actin flow. **c** Average F-actin flow speed as a function of the gel stiffness of SMIFH2-BBS-treated cells (red, n=8, 11, 12, 14, 9 cells for increasing stiffness, collected from m=57937, 42861, 79091, 99196, 80533, 54022 windows). **d** Representative interpolated flow vectors (top) and speed maps (bottom) of SNAP-actin of SMIFH2-BBS-treated fibroblasts on 0.6 kPa, 1.3 kPa, 2.6 kPa, 6 kPa and 12.7 kPa gel. Arrow scale: 1 μm min^−1^ of actin flow. **e** Average F-actin flow speed as a function of the gel stiffness of LatA-BBS treated cells (red, n=9, 10, 8, 8, 12 cells for increasing stiffness, collected from m=52341, 52948, 36332, 45756, 65410 windows). A negative exponential function ($V=a*\text{exp}(R_{0}E)+V_{0}$) was used for flow speed vs stiffness plots in (**a**), (**c**) and (**e**) (See Supplementary Table 2 for fit parameters). **f** Representative interpolated flow vectors (top) and speed maps (bottom) of SNAP-actin of LatA-BBS-treated fibroblasts on 0.6 kPa, 1.3 kPa, 2.6 kPa, 6 kPa and 12.7 kPa gel. Arrow scale: 1 μm min^−1^ of actin flow. scale bar: 10 μm. **g** Normalized actin speed of BBS (white) and CK666-BBS (yellow) treated cells for each corresponding stiffness. *: *p* < 0.05, **p < 1×10^−20^, ***p < 1×10^−30^ by Mann-Whitney U test. **h** Normalized actin speed of CK666-BBS- (yellow), SMIFH2-BBS- (dark grey) and LatA-BBS-inhibited cells (light grey). *p < 0.05, **p < 1×10^−20^, ***p < 1×10^−30^ by Kruskal-Wallis ANOVA test with Dunn’s post hoc analysis. **i** A plot of curve fits of actin flow speed as a function of the stiffness for all conditions for comparison purpose.

In order to investigate whether the decrease in stiffness-dependent traction observed in cells with actin polymerization inhibition is linked to actin retrograde flow, we measured the velocity of F-actin flow in cells subjected to double inhibition with CK666-BBS (Supplementary Movie 3), SMIFH2-BBS (Supplementary Movie 4), or LatA-BBS (Supplementary Movie 5) using qFSM. Cells treated with CK666-BBS exhibited a decline in actin flow speed with increasing stiffness in the range of small-to-intermediate stiffness (0.6 kPa to 2.6 kPa), followed by a plateau in higher stiffness (6 kPa to 12.7 kPa) (Fig. 3a). This trend resembled the flow pattern observed in myosin-II-inhibited cells (Fig. 1g), with a slightly lower flow speed that was not statistically significant (Fig. 3g). This indicates that the traction trend in cells with Arp2/3 and myosin-II inhibition is dependent on flow. Most of the high flow was concentrated at the periphery of the cell (Fig. 3b), similar to myosin-II-inhibited cells. Conversely, double inhibition of myosin-II and formin with SMIFH2-BBS treatment greatly reduced the flow, leaving only minimal stiffness dependency in the low stiffness regime (Fig. 3c). Additionally, cells treated with LatA-BBS exhibited a consistent flow speed across all stiffness values (Fig. 3e, f, h). Overall, the flow speed was profoundly reduced in cells treated with SMIFH2-BBS and LatA-BBS compared to CK666-BBS-treated cells (Fig. 3h). The gradual decrease in stiffness-dependence of actin flow speed, progressing from BBS-only to CK666-BBS to SMIFH2-BBS to LatA-BBS (Fig. 3i), aligns with the gradual reduction in differential traction observed (Fig. 2i). These flow results collectively suggest a strong association between the reduced stiffness-dependent force transmission and actin’s contribution to retrograde flow.

### Traditional molecular-clutch model assuming rigid F-actin flow alone cannot explain Arp2/3-dependent, myosin-II-independent flow-traction behaviors.

Stiffness-dependent traction transmission has been explained by a molecular clutch model where traction is transmitted to the ECM by a dynamic clutch between integrin-based adhesion complex against a flowing F-actin^18,21,22^. In the model, a higher traction is transmitted against stiffer ECM because the traction develops in a faster rate if the clutch engagement is stable^21^. Another contributing component has been myosin-II that pulls F-actin with a muscle-like behavior, *i.e.,* an inverse relationship between the flow velocity and the force in the fiber^64-66^. For example, the same amount of force pulls the stiffer ECM with less deformation. A slower deformation rate $v_{\text{myo}}$ against a stiffer ECM gives rise to a higher force $F_{\text{myo}}$ according to the force-velocity relationship (*e.g.*, a linearized inverse relationship such as in ($F_{\text{myo}}=F_{\text{stall}}(1-v_{\text{myo}}∕v_{o})$). However, this model relied solely on myosin-II as a force generator and thus was not able to explain our traction data (Figs. 1 and 2) with myosin-II inhibition.

**Fig. 4:**
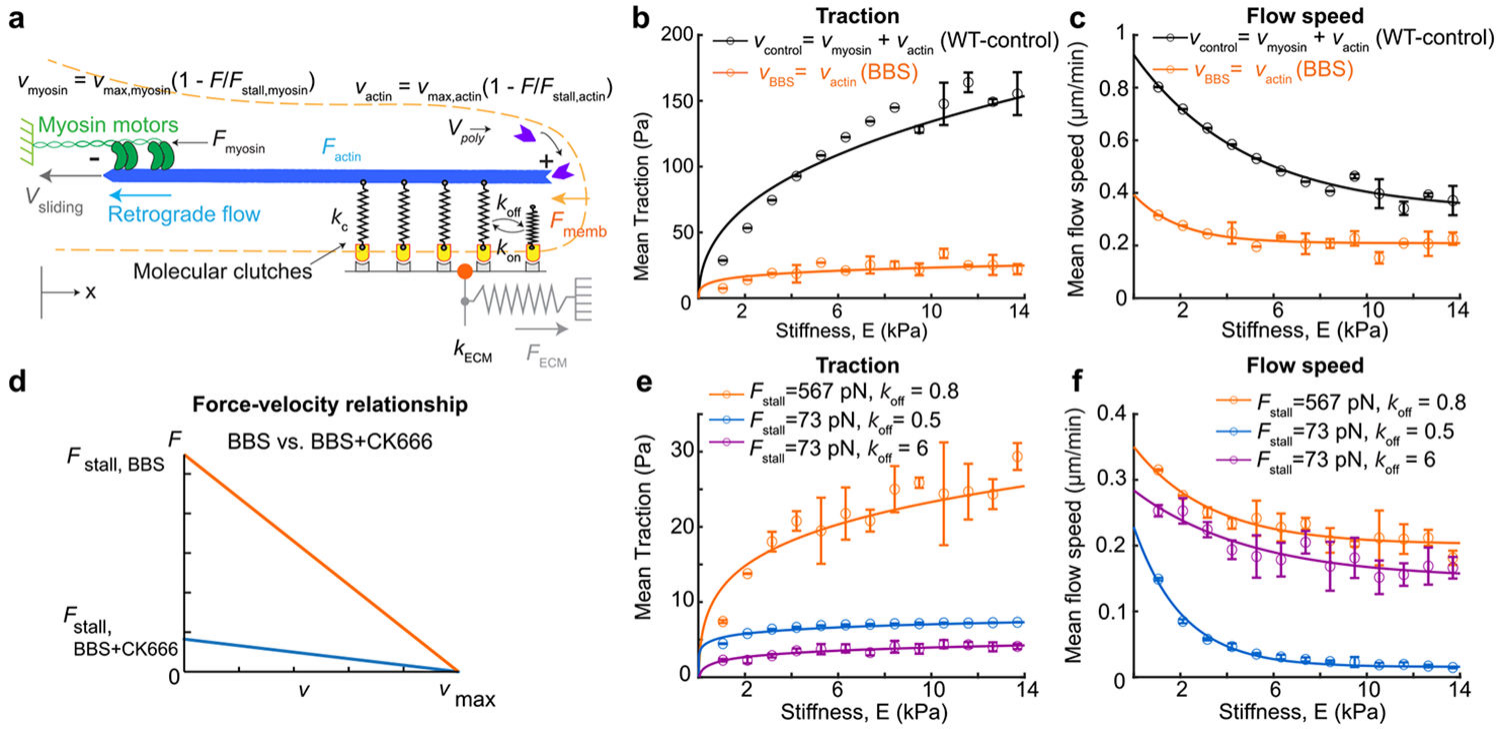
A molecular clutch model assuming rigid actin flow insufficiently explains Arp2/3′s role in stiffness-dependent force-flow behaviors. **a** A schematic of the molecular clutch model modified with addition of actin polymerization. Actin polymerization-powered retrograde flow velocity as a function of force, $v_{\text{actin}}=v_{\max,\text{actin}}\left(1-F∕F_{\text{stall},\text{actin}}\right)$, was added to the myosin motor-generated actin retrograde flow velocity, $v_{\text{myosin}}=v_{\max,\text{myosin}}\left(1-F∕F_{\text{stall},\text{myosin}}\right)$. Myosin-independent, stiffness-dependent traction was simulated by considering only $v_{\text{actin}}$ without $v_{\text{myosin}}$ (see materials and methods for details). **b** Simulated results of traction as a function of a substrate stiffness. **c** Simulated results of actin flow speed as a function of a substrate stiffness. WT-control (*black*) was simulated using both $v_{\text{myosin}}+v_{\text{actin}}$ but BBS (*orange*) using only $v_{\text{actin}}$. Traction data in (**b**) were fitted using a power law function, i.e., $f=aE^{b}$ where a=60.8 and $b=0.38\times 10^{-10}$ for WT-control and $a=6.65_{0}$ and $b=0.14\times 10^{-10}$ for the BBS condition. Flow speeds in (**c**) were fitted using a negative exponential relationship, $V=a*\text{exp}(R_{0}E)+V_{0}$ where a=0.60, $R_{0}=-0.21$, $V_{0}=0.33$ for WT-control and a=0.18, $R_{0}=-0.50$, $V_{0}=0.21$ for the BBS condition. **d** A force-velocity relationship of actin-polymerization-powered retrograde flow, modeled for actin with (*orange*) and without (*blue*) Arp2/3. Lower stall force ($F_{\text{stall},\text{CK}666}$) was assumed for a flow in cells without Arp2/3 and myosin activities than the one ($F_{\text{stall},\text{BBS}}$) with Arp2/3. The stall force was modeled to be a function of a local actin density, $d_{\text{actin}}$. **e**, **f** Simulated results of traction (**e**) and actin retrograde flow speed (**f**) as a function of stiffness in BBS (*orange*) and CK666-BBS conditions (*blue, magenta*). CK666-BBS (*blue*) were simulated using lower actin stall force than BBS condition, *i.e.,* $F_{\text{stall},\text{CK}666}<F_{\text{stall},\text{BBS}}$ while having low off-rate, $k_{\text{off}}=0.8$, for clutch binding. CK666-BBS (*purple*) traction and retrograde flow were simulated with high off-rate, $k_{\text{off}}=6$. Traction data was fitted using power law curve, $f=aE^{b}$, where a=0.2, $b=0.3\times 10^{-10}$ for BBS; a=15.3, $b=0.05\times 10^{-10}$ for CK666-BBS; a=12.2, $b=0.06\times 10^{10}$ for CK666-BBS-slip. Flow speed was fitted using a negative exponential relationship, $V=a*\text{exp}(R_{0}E)+V_{0}$ where a=0.61, $R_{0}=-0.22$, $V_{0}=0.33$ for BBS; a=0.21, $R_{0}=-0.48$, $V_{0}=0.02$ for CK666-BBS (*blue*); a=0.15, $R_{0}=-0.30$, $V_{0}=0.20$ for CK666-BBS-slip (*purple*).

To explain myosin-II-independent, stiffness-dependent traction trend, we added actin polymerization-powered retrograde flow velocity to the model (Fig. 4a and Materials and Methods). The polymerizing F-actin can create not only edge protrusion but the retrograde flow by being pushed back by the membrane^22,65^. When both edge protrusion speed and actin flow speed were quantified from the individual cells, we found that the retrograde flow speed was proportional with cell protrusion speed in response to gel stiffness (Supplementary Fig. 4). These measured observations support the notion that actin polymerization gives a balanced contribution to both membrane protrusion and actin retrograde flow. Thus, for the model, we assumed that the actin polymerization-powered flow velocity $v_{\text{actin}}$ is proportional with the actin polymerization rate and ignored the edge movement. Importantly, in-vitro experiments^67,68^ and physics models^69,70^ have found that polymerizing actin also exhibits an inverse relationship between the protrusion force and the polymerization rate. Accordingly, we modeled actin-polymerization-powered retrograde velocity $v_{\text{actin}}$ as an inverse function (but linearized) of a force (Fig. 4a), as done similarly for $v_{\text{myo}}$.

To simulate the myosin-II-inhibited, stiffness-dependent traction response, we forced $v_{\text{myo}}$ to be zero, which left only $v_{\text{actin}}$ active, while both velocities were alive for the simulation of WT cell traction behavior. As suggested from in-vitro measurements^71-73^, we assumed the stall force for F-actin polymerization to be near one-third of the stall force by myosin. This simulation was able to recapitulate the stiffness-dependent traction trend (Fig. 1a, c) by showing overall diminished force magnitude by BBS-treated cells compared to those by WT cells but a still differentially increasing traction trend in a stiffness-dependent manner (Fig. 4b). Similarly, the same simulation resulted in the inverse trend of actin flow speed as a function of the stiffness for both WT and BBS-treated cases with 3-fold lower speed in BBS-treated cell cases than the one for WT cell cases (Fig. 4c), recapitulating the experimental findings (Fig. 1e, g). This could be understood on the framework of the traditional molecular clutch model^21^ except for the added $v_{\text{actin}}$. Briefly, the F-actin network on a soft substrate flows faster because the tension develops slower owing to the substrate compliance, which allows still a large velocity according to the force-velocity relationship ($F_{\text{actin}}=F_{\text{stall}}(1-v_{\text{actin}}∕v_{o})$). But due to the limited lifetime of the clutch linking both F-actin and the ECM, the substrate deforms finitely, thus transmitting still small traction, (Supplementary Fig. 5a). On a stiffer substrate, the tension develops at a faster rate, which results in smaller velocity from the force-velocity relationship, thus resulting in more frequent clutch unbinding but still higher traction (Supplementary Fig. 5b).

Next, we attempted to predict the traction-and-flow behaviors exhibited by cells with Arp2/3 and myosin-II inhibitions. Our experimental data showed that the traction magnitude in myosin-II-inhibited cells was nearly 4 times larger than Arp2/3-myosin-II-inhibited cells (Fig. 2i, *red vs. green*) while the flow speed differed by only ~20 % (Fig. 3i, *red vs. green*). To recapitulate this seemingly excessive difference, we simulated our model with a lower stall force ($F_{\text{stall}}$) for CK666-BBS case in the actin’s force-velocity relationship (Fig. 4d), with a rationale that the polymerizing actin could bear much smaller force without Arp2/3 than one with it. This input indeed lowered the traction by 4-fold compared to the BBS-only condition setting (Fig. 4e, *orange vs. blue*). However, the simulation led to an actin flow speed much lower than that from BBS-only setting (Fig. 4f, *orange vs. blue*). The reason why the flow reaches near zero was because we didn’t allow the adhesion unclutching by elevating $k_{\text{off}}$ rate constant. To allow more unclutching events, we lowered $k_{\text{off}}$ value in the model, and this model was able to simulate high-enough actin flow speed compared to BBS-only model (Fig. 4f, *orange vs. purple*). However, this change led to further reduction in traction as well (Fig. 4e, *orange vs. purple*), resulting in too low traction magnitude overall. Together, the traditional clutch model partially explains myosin-II-independent, stiffness-dependent differential traction but contains a limit when it comes to contributions from actin nucleators.

### Considering F-actin elasticity can explain the mechanosensitive roles of actin nucleators.

We identified a key issue with the traditional model, namely, its assumption of F-actin as a completely rigid entity, where its motion was solely governed by the force-velocity relationship rather than considering force balance and constitutive equations such as Hooke’s law. In that setting, if the actin-clutch binding-unbinding dynamics are similar, it becomes difficult for the flowing actin to transmit considerably different force levels from similar flow velocity levels. To overcome this difficulty, we considered the F-actin’s intrinsic viscoelastic properties. F-actin displays viscoelasticity across multiple scales^62,74^, a characteristic that has been substantiated through in-vitro studies where the concentration of actin monomers^75^, or the addition of Arp2/3 has been shown to enhance the elasticity of purified actin gels^76^. This observation underscores our hypothesis that the polymerization of F-actin, which is influenced by nucleation, branching, and the presence of cross-linking proteins, have a cumulative effect on the mechanical behavior of the F-actin network. In the context of the lamellipodium, although individual actin filaments may be shorter than their persistence length and, therefore, considered mechanically rigid when isolated, they collectively form a network. The overall elasticity of this network is not merely a function of the rigidity of single filaments; it is also determined by the interplay between filament density and the dynamics of their cross-linking. This complex interdependence means that a softer F-actin network would transmit less force in response to a given displacement, highlighting the critical role of network architecture in cellular force transduction.

**Fig. 5:**
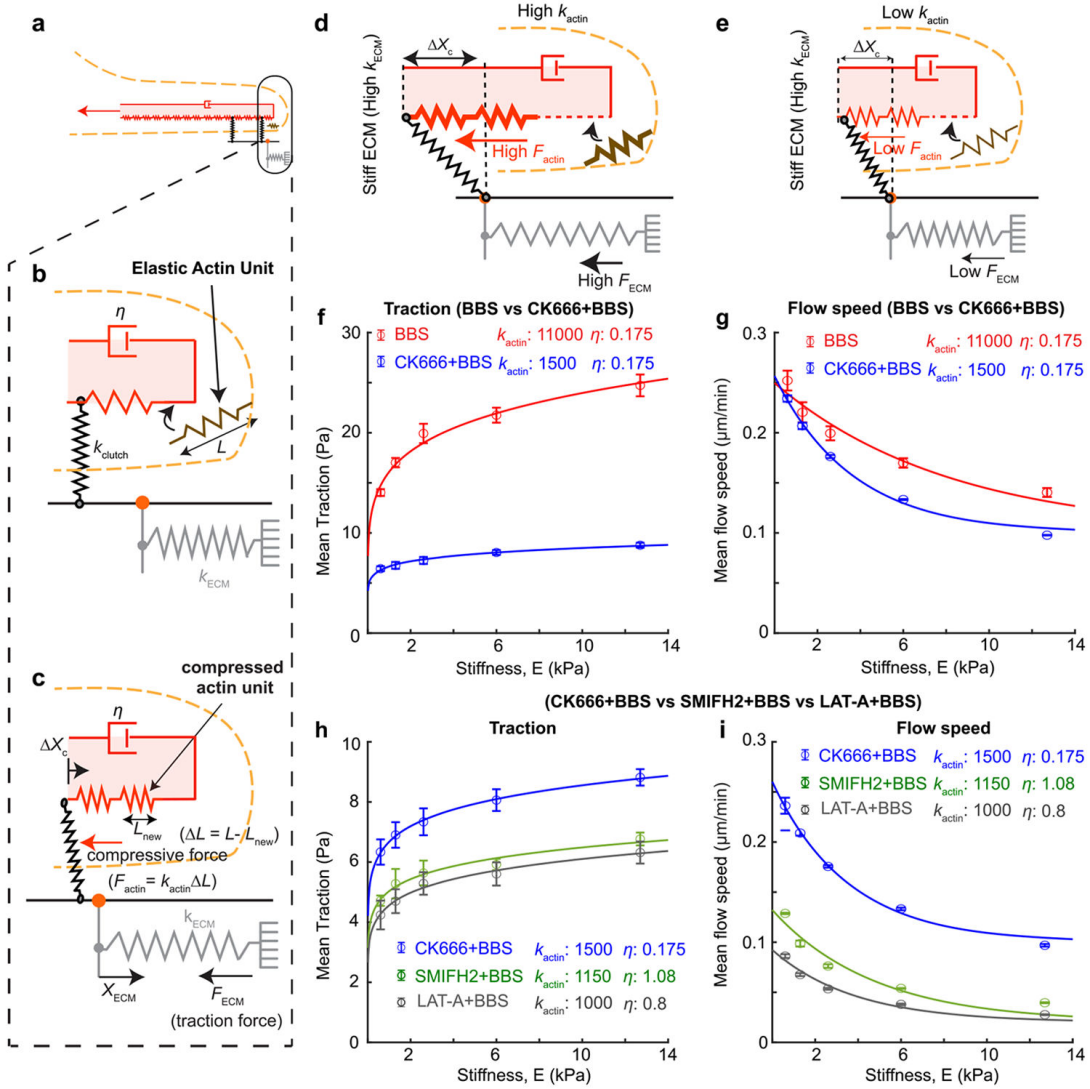
Actin-elasticity-based, actin-polymerization-powered molecular clutch model. **a** An overview of the new molecular clutch model that models F-actin as a viscoelastic material. **b, c** Magnified views of a cell leading edge before (**b**) and after (**c**) addition of an elastic actin unit at a polymerizing tip. The elasticity of an elastic actin unit, $K_{\text{actin}}$, is an meso-scale modulus and comes from the architecture and morphology of actin fiber network in addition to thickness of individual fibrils and fibers. The added actin unit leads to compressive force, $F_{\text{actin}}=k_{\text{actin}}\Delta L$, by the boundary conditions at the membrane tension (*right*) and at the clutch (*left*) where ΔL is the change in length of individual actin units. The membrane was assumed to be a rigid wall (see Supplementary Fig. 4 for justification). The compressive force displaces the clutch and is transmitted to the substrate as traction, $F_{\text{ECM}}$. The displacement and the force balance are damped by a viscous damper, η, in the actin. **d, e** Illustration that compares the model with high (**a**) vs. low (**b**) actin elasticity, $k_{\text{actin}}$. **d** With high $k_{\text{actin}}$, addition of a new actin unit results in high force, $F_{\text{actin}}$, which leads to high _c_lutch displacement, $\Delta x_{c}$, and thus high traction, $F_{\text{ECM}}$. **e** With low $k_{\text{actin}}$, however, addition of the same original length of the actin unit creates only small $\Delta x_{c}$ and small $F_{\text{ECM}}$ because it is compressed more easily. **f, g** Model prediction for traction (**f**) and retrograde flow speed (**g**) of BBS- (*red*) and CK666-BBS-treated cells (*blue*). Note that the only difference between the two conditions is $k_{\text{actin}}$, 11,000 (BBS) vs. 1500 (CK666-BBS). Traction data was fitted using power law curve, $f=aE^{b}$, where a=16.098 and b=0.173 for BBS and a=6.650 and b=0.106 for CK666-BBS. Flow speed was fitted using a negative exponential relationship, $V=a*\text{exp}(R_{0}E)+V_{0}$ where a=0.150, $R_{0}=-0.123$, $V_{0}=0.10$ for BBS and a=0.157, $R_{0}=-0.227$, $V_{0}=0.10$ for CK666-BBS. **h, i** Model prediction for traction (**h**) and retrograde flow (**i**) of CK666-BBS- (*blue*), SMIFH2-BBS-(*green*) and LatA-BBS-treated cells (*black*). CK666-BBS was simulated using $k_{\text{actin}}=1500$ with a viscosity η=0.175. SMIFH2-BBS and LatA-BBS conditions were simulated using $k_{\text{actin}}=1150$ and η=1.08, and $k_{\text{actin}}=1000$, viscosity η=0.8, respectively. Traction data was fitted using a power law curve, $f=aE^{b}$, where a=6.675, b=0.108 for CK666-BBS; a=5.032 and b=0.110 for SMIFH2-BBS; a=4.561 and b=0.126 for LatA-BBS. Flow speed was fitted using a negative exponential relationship, $V=a*\text{exp}(R_{0}E)+V_{0}$ where a=0.160, $R_{0}=-0.284$, $V_{0}=0.1$ for CK666-BBS; a=0.112, $R_{0}=-0.211$, $V_{0}=0.002$ for SMIFH2-BBS; a=0.072, $R_{0}=-0.261$, $V_{0}=0.02$ for LatA-BBS.

To test this idea, we modeled the polymerizing actin as a viscoelastic continuum material in a scale large enough to contain actin-binding proteins as well as actin itself (Fig. 5a). At the polymerizing tip of the model, the F-actin network grows by addition of discrete elastic actin unit with length L, of which the viscoelasticity is determined in mesoscale, *i.e.,* by an integrative effect from the 3D actin architecture and molecular interactions (Fig. 5b) (see Materials and Methods for details). The addition of new actin unit compresses the 1D actin network which is bounded by individual adhesion clutches. The compressed elastic force is transmitted to the clutches, which is again transmitted to the elastic substrate as a traction force, $F_{\text{ECM}}$ (Fig. 5c). As more F-actin units are added, the more compressive force progressively loads either slowly or quickly depending on the substrate stiffness or the actin elasticity. The addition of the actin unit is controlled by the level of compression of spring, i.e., if the actin springs are densely packed, less actin units can be added (Eq. $N_{\text{new}}=N_{\mathrm{nmax}}-\frac{N_{\mathrm{nmax}}}{F_{s,\text{actin}}*F_{c,\max}}$). Upon release of clutch from the ECM due to high-force and slip-bond adhesion kinetics, the compressed actin units relax by expansion, creating a quick retrograde flow, dampened by actin viscosity (Fig. 5c, *a damper with a coefficient* η).

A key difference of this new model compared to the previous traditional model is that the F-actin elasticity ($k_{\text{actin}}$) directly affects the transfer of the force. For example, the same addition rate of a new actin unit can create quite different traction transmission: the addition of stiff actin unit can pull the clutch and the substrate much further (Fig. 5d) than that of soft actin (Fig. 5e). Indeed, via changing values of only the actin elasticity $k_{\text{actin}}$, from $k_{\text{actin}}=11000$ for BBS to $k_{\text{actin}}=1500$ for CK666-BBS case, the new model was able to recapitulate the experimental traction and flow data between BBS-treated vs. BBS-CK666-treated cells, *i.e.,* by exhibiting ~4-fold difference in traction (Fig. 5f) but ~20% difference in actin flow speed (Fig. 5g). This suggests that the consideration of Arp2/3 to F-actin elasticity is important for the force-sensitivity to the ECM stiffness.

Not only Arp2/3, but formin’s actin nucleation activity contributes to the elasticity of the actin filamentous network^77^ via modulating actin filament length^62^. Actin cortex elasticity is also reduced by LatA treatment^78,79^. To seek if the lowered traction and actin flow could be explained by F-actin network elasticity changes, we simulated traction and retrograde flow of cells treated with SMIFH2-BBS and LatA-BBS using $k_{\text{actin}}$ as the main variable. Interestingly, by further lowering $k_{\text{actin}}$ with slight increase in viscosity η, the model was able to recapitulate the stiffness-dependent differential tractions (Fig. 5h) with much less (for SMIFH2-BBS-mimicking simulation with $k_{\text{actin}}=1150$ and η=1.08) or near-flat (for LatA-BBS-mimicking simulation with $k_{\text{actin}}=1000$ and η=0.8) actin flow speed in response to the ECM stiffness (Fig. 5i). Together, our new model simulation results demonstrate that the viscoelasticity of the network plays an important role in the myosin-II-independent mechanosensing response.

### Arp2/3 contributes to the F-actin network elasticity, formin contributes to it more.

**Fig. 6:**
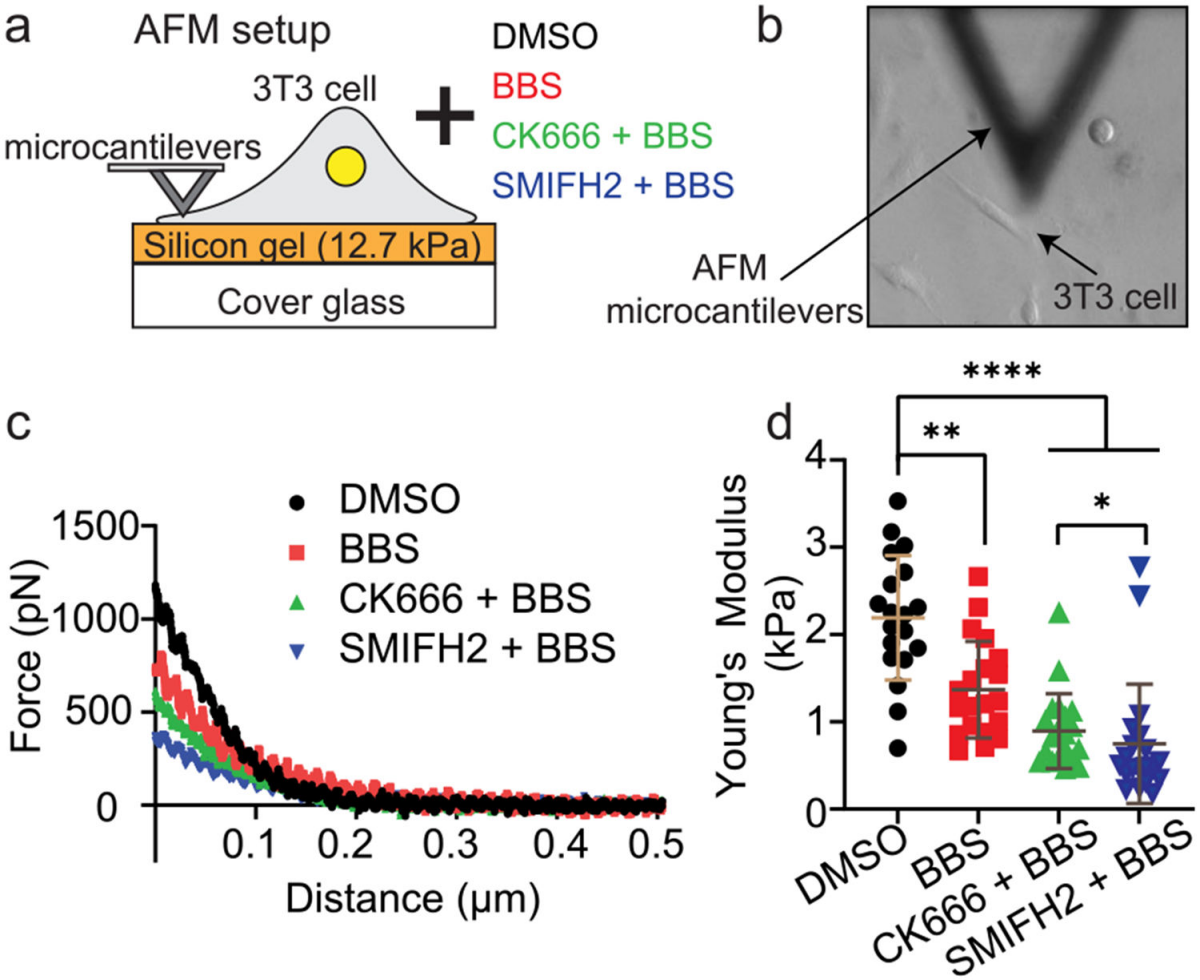
F-actin elasticity decreases with inhibition of Arp2/3 and formin. **a** A schematic of live cell atomic force microscopy (AFM) force spectroscopy using a modified AFM microcantilever with an attached 10 μm spherical probe in WT-control (DMSO), BBS, CK666-BBS and SMIFH2-BBS conditions. **b** A top view brightfield image with a triangular AFM microcantilever and spread NIH-3T3 fibroblasts. **c** Representative force-distance curves after the contact between cell and AFM microcantilever tip in WT-control and inhibitor conditions. The force curve was fitted using Hertz contact mechanics to calculate Young’s modulus. **d** Young’s modulus of the cell as a function of different conditions calculated from the force-distance curve. N=20 cells for each condition. Mean ± S.D. *p < 0.05, **p < 0.01, ***p < 0.001, ****p < 0.0001.

To validate our new model assumption that Arp2/3 and formin contribute to the viscoelasticity of the F-actin network, we used live-cell-probing atomic force microscopy (AFM) to measure the lamellipodium F-actin network elasticity of 3T3 fibroblasts plated on a gel with 12.7 kPa stiffness (Fig. 6a). The pharmacological conditions were kept consistent with those used in TFM and qFSM experiments, *i.e.,* WT control, BBS, CK666-BBS, and SMIFH2-BBS. In order to assess the F-actin network elasticity, AFM force spectroscopy was done in the lamellipodium region of the spread fibroblasts using a modified microcantilever attached with a 10-μm-diameter spherical probe^80^ (Fig. 6a, b). For cells per condition, AFM force-distance curves were obtained by applying a set force ranging between 400 pN - 1.2 nN, which results in indentation between 100 nm and 200 nm, deep enough to sense actin network elasticity (Fig. 6c). Subsequently, Young’s modulus of the cell was calculated by fitting these indentation curves into the Hertz model (outlined in detail in Materials and Methods section). BBS-treated cells showed a significant reduction in F-actin elasticity compared to WT cells (Fig. 6d), which is consistent with a previous finding with 3T3 and NRK fibroblasts^81^. Furthermore, inhibition of Arp2/3 activity along with myosin-II (CK666-BBS treated cells) reduced the F-actin network elasticity even further than BBS-treated (~0.5-fold decrease) and WT cells (~2.5-fold decrease) (Fig. 6d). We also observed that cells treated with SMIFH2-BBS displayed further lowered F-actin elasticity compared to CK666-BBS-treated cells (Fig. 6d). These results support the assumption in our model that Arp2/3 and formin activities elevate the F-actin network elasticity with higher impact by formin than Arp2/3.

The reduction in the F-actin network elasticity of cells treated with CK666-BBS compared to BBS-treated cells was ~65% (Fig. 6d), which was much smaller than our assumed reduction (~10.5%) in the model (Fig. 5f, g). Accordingly, we used less reduction (36 % and 64%) in the F-actin elasticity ($k_{\text{actin}}$) in our model to simulate the traction and the actin flow of cells treated with CK666-BBS (Supplementary Fig. 6). This simulation showed a trend similar to the previous result with 10.5% reduction, i.e., significant reduction in traction and small change in the flow speed (Supplementary Fig. 6a, b compared to Fig. 5f, g). Simulations with the less-reduced F-actin elasticity required the elevation of the viscosity part of the actin viscoelasticity to dampen the flow speed. Thus, the AFM data not only validated our model assumption, but also provided more accurate quantitative reference for model prediction.

### Frequency of F-actin flow speed increases with ECM stiffness as the new model predicts.

**Fig. 7:**
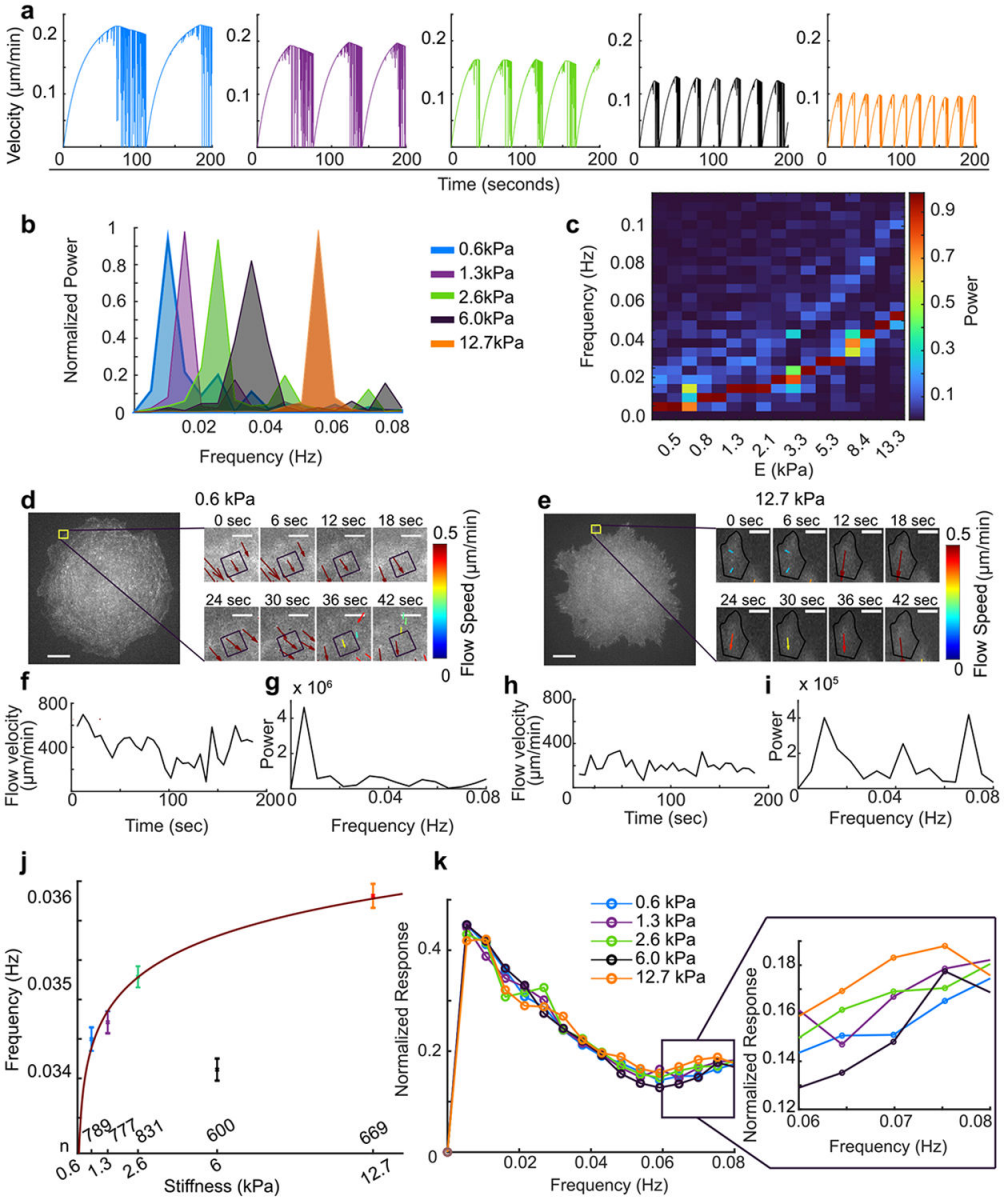
Both model and experiments show that actin flow speed frequency increases with the substrate stiffness. **a** Actin flow velocity simulated using the actin-elasticity-based clutch model as a function of time on substrates with increasing stiffness, i.e., 0.6 kPa (*blue*), 1.3 kPa (*purple*), 2.6 kPa (*green*), 6.0 kPa (*black*) and 12.7 kPa (*orange*). Note that more frequent unclutching events, represented by velocity drop to zero, occurs as the stiffness increases. **b, c** Frequency analysis of the simulated time-series of velocities with a power spectrum distribution plot (**b**) and 2D-histogram of frequency power as a function of stiffness (**c**). **d, e** Representative images of SNAP-SiR647 actin in 3T3 fibroblasts treated with BBS on soft (0.6 kPa) (**d**) and stiff (12.7 kPa) substrates (**e**). *Right:* Montage of SNAP-actin over time with color-coded flow vectors in the yellow-boxed window on the full image. **f, g, h, i** Time-series plots of flow velocities (**f, h**) and power of the flow frequency (**g, i**) of a cell on a 0.6 kPa gel (**f, g**) vs. on a 12.7 kPa gel (**h, i**), sampled from 1×1 μm window in **d** and **e**, respectively. **j** Average frequencies of hundreds of windows of multiple cells as a function of stiffness. The numbers of windows, n, are denoted on top of x-axis. The numbers of cells per stiffness are: 7, 9, 9, 9, 9 cells for increasing stiffness. A power law curve, $f=aE^{b}$, was used to fit the observed data, where $a=0.003469\pm (3.5\times 10^{-4})$ and b=0.01767±0.0073 with $R^{2}=0.9817$. The 4^th^ data point was excluded as an outlier, i.e., outside of 1.5 standard deviation of the output data. **k** Normalized power spectra of all windows of cells in all five stiffness conditions. *Right*: Zoomed-in view of the normalized power spectra in high-frequency regime (0.06–0.08 Hz). Note the higher power of high stiffness-related actin flow frequency (e.g., 12.7 kPa) than low stiffness-related actin flow frequency in the high-frequency regime.

Our model predicts that during the clutch engagement, the F-actin unit addition induces minimal F-actin displacement while the unclutching event leads to the rapid expansion of the compressed actin network, thus exhibiting high flow speed. Accordingly, cycles of clutching and unclutching could create fluctuations in F-actin flow speed. As the force builds up faster on a stiff ECM, the potential fluctuation could become also faster. Indeed, our model simulation with the same off rate of the clutch predicted that the stiffer the substrate is, the more often the clutch is released and thus allows more frequent actin flow speed fluctuations (Fig. 7a). Power spectrum analysis of the frequencies of the simulated time series has confirmed this stiffness-dependent flow speed frequency (Fig. 7b, c). To identify whether this flow characteristics is present in fibroblasts, we analyzed the actin flow field of BBS-treated cells by sampling from finite-sized windows, 1 μm by 1 μm, along the cell perimeter of a cell area. An example flow velocity in a window of a cell on a soft (0.6 kPa) substrate (Fig. 7d) was high overall (Fig. 7e) but the transformation into a frequency domain showed that it exhibited a low-frequency spectrum overall with the majority of power in a low (0.005–0.01 Hz) frequency regime (Fig. 7f). In contrast, a small flow vector found in a cell on a stiff (12.7kPa) substrate exhibited more fluctuation compared to the overall magnitude (Fig. 7g, h). Analyzing the frequency spectrum showed significant power not only in a low-frequency regime but also in a high-frequency (0.04–0.08 Hz) regime (Fig. 7i). Indeed, the average frequencies collected from several cells and hundreds of windows showed an increase as a function of the stiffness (Fig. 7j, *p* < 8 ×10^−6^ from power-law fit). Unlike the simulation, normalized power spectra of all windows appeared similar among all stiffness conditions (Fig. 7k). However, there were higher powers in cells on stiffer substrates in the high-frequency regime, e.g., 0.06–0.08 Hz (Fig. 7k, inset), which led to the difference observed in the average frequency (Fig. 7k). Together, this quantification further validates the actin elasticity-based molecular clutch model for actin-based rigidity sensing.

## Discussion

In this study, we provide a stiffness-sensing mechanism for adherent cells when myosin-II, a primary force generator in a cell, is not actively working. Our model suggests that actin nucleators control the sensitivity for stiffness-dependent differential force transmission by modulating the elasticity of the polymerizing actin. Our data demonstrate that the retrograde flow, which still exists in the absence of myosin-II activity via actin polymerization^32,52,82^, is able to induce the stiffness-dependent differential transmission. Actin polymerization has been well established for its contribution to cell migration^83,84^ or cell shape determination^85^, mostly in keratocytes, or to the generation of the pushing force and corresponding edge-protrusion^68,69,86^. However, this actin polymerization-based force has shown to be independent of substrate stiffness^67,73^. Our work suggests that only after combining the actin polymerization-generated flow with the clutch dynamics, the stiffness-dependent differential traction can emerge. Previous molecular clutch models^18,22^ incorporated parameters for actin polymerization, but only they were not considered to be the primary source of the retrograde flow and traction forces. Our model, for the first time to the best of our knowledge, uniquely integrates actin polymerization and viscoelasticity, serving as dual contributors to both actin flow and traction forces, which, in conjunction with clutch dynamics, are modulated by substrate stiffness. This approach delineates cellular mechanics without relying on myosin-II-driven forces.

Our results show that actin flow decreases upon inhibition of myosin-II and Arp2/3, which is consistent with those reported with neurons^82^. In that study, upon Arp2/3 inhibition, an even faster actin flow has been reported than one by control WT neuron cells^82^. Only after additional inhibition of myosin-II the flow speed became much smaller than WT cells^82^. Their results imply that Arp2/3-mediated branched actin meshwork resists against myosin-II-generated F-actin flow, without which the flow can be even more accelerated. Our data suggest that if myosin-II activity is inhibited, Arp2/3 actively contributes to retrograde flow generation, and without Arp2/3 activity actin flow speed is reduced. We speculate that the reason for accelerated flow with Arp2/3-only inhibition is Arp2/3’s involvement with nascent adhesions potentially through vinculin, providing friction against flowing actin^87-89^. Considering Arp2/3-adhesion coupling, it is possible that the reduced traction in cells with CK666-BBS is in part attributed to weaker adhesivity triggered by Arp2/3 inhibition, in addition to less F-actin network elasticity we presented here.

In addition, our data demonstrated that formin endows more mechano-sensitivity to cell-ECM adhesions than Arp2/3 across all stiffness tested. This result could be recapitulated by lowering the elasticity of the _F-actin in the case of formin inhibition compared to Arp2/3 inhibition in the new model. Why and how formin contributes to F-actin network elasticity more strongly than Arp2/3 is not clearly understood. One idea is that formin contributes to the actin elasticity by controlling the average filament length. Formin inhibition by SMIFH2 has shown to decrease the length of long F-actin filaments^61,77,90^, whereas Arp2/3 inhibition increases the average F-actin length^91^. An in-vitro reconstitution study using purified F-actin and a capping protein gelsolin has shown that the longer the F-actin filament is, the stiffer the F-gel becomes^62^. At the first look, F-actin stiffening by filament lengthening appears counterintuitive. A traditional bio-polymer model, also known as an affine model, predicts that the distance between crosslinks negatively controls the elastic modulus^92^. A similar model combined with cell membrane predicts that long F-actin filaments cannot withstand a large force because they bend under the force whereas shorter filaments can transmit forces much more efficiently^93^. These models thereby might not be able to explain the proportional relationship between the F-actin length and the actin elasticity. As an alternative, the length-dependent F-actin stiffening has been explained by a model of rigid polymers connected by flexible crosslinks^62,94,95^. In this model, a longer filament can accommodate the greater number of flexible crosslinks along its length, which allows the gel to withstand a larger load^62^. A further developed mathematical model, referred to as ‘hairy rod model’, predicts that an increase in filament length results in not only stiffening but also temporary softening when the length increases beyond twenty times of the filament radius^96^. Together, formin might contribute to the F-actin elasticity and thereby the mechanosensitivity by increasing the average filament length but up to small length scale.

In the presence of BBS and LatA, there is still an increase in traction with stiffness that cannot be explained by actin polymerization. For potential myosin-II and F-actin-independent mechanisms that could contribute to the observed increase in traction with substrate stiffness, we considered the roles of both membrane tension and cytoskeletal prestress outside of F-actin network. Two recent studies have shown that both membrane tension^97^ and cortical stiffness^98^ are not directly dependent on substrate stiffness, suggesting that the membrane tension is not a critical component that endows cells actin-independent, myosin-II-independent, stiffness-dependent differential traction. However, the prestress within the cytoskeleton is still held as a promising source. The prestress could be contained in passive elastic elements within the cell that are engaged upon deformation, or alternative force-generating mechanisms such as those mediated by microtubules (MTs) or intermediate filaments (IFs), which are not directly affected by BBS or LatA^99^. This possibility is opposed by a conventional tensegrity model where the microtubule is responsible for resisting compression, not tension^100^, thus it requires further testing with inhibitors of IFs or MTs. Furthermore, the complexity of cell-matrix adhesion dynamics provides another dimension to stiffness sensing. These adhesions, through catch-bond behavior and mechanosensitive signaling pathways, retain the capacity to sense and transduce mechanical signals in the absence of actin dynamics. These insights emphasize the multifaceted nature of cellular mechanotransduction, extending beyond the actomyosin architecture, and warrant a broader consideration of the myriad components that contribute to mechanosensitivity.

Previous studies have established that fibroblasts adapt their own elasticity to match the stiffness of the ECM^101^. The elasticity of the F-actin cytoskeleton, which contributes significantly to the cell’s cortex elasticity, is influenced by passive crosslinkers such as Arp2/3 and formin^102^ as well as active force-generating crosslinkers like myosin^48^. Both in vitro reconstituted systems^103,104^ and in vivo experiments^105^ have demonstrated that branched F-actin networks are mechanosensitive, exhibiting an increase in branching density in response to higher loads. However, whether the regulation of Arp2/3 or formin in response to ECM stiffness directly affects F-actin network elasticity, to the best of our knowledge, has yet to be investigated. Nevertheless, our model, which assumes a constant F-actin elasticity for different ECM stiffness levels under a given perturbation condition, successfully replicates the stiffness-dependent differential traction observed. This finding suggests that clutch dynamics, driven solely by actin polymerization, enable cells to perceive higher stiffness by transmitting increased traction, even with the same F-actin elasticity. Thus, the adaptation of cell elasticity to ECM mechanics may involve not only actin crosslinkers but also clutch dynamics mediated by both actin polymerization and myosin-II contractility.

Our actin elasticity-based molecular clutch model provides a possible explanation for more frequent F-actin flow speed fluctuation coupled to higher substrate rigidity. The stiffness-dependent edge contraction have been reported previously in mouse embryonic fibroblasts, which depends on activities of myosin, cofilin and Rac1^106^. We believe that the reason why we observe the stiffness-dependent flow speed fluctuation is owing to a large data sampling followed by a detailed frequency analysis. Together, our simulation and experimental results suggest that the F-actin’s elastic compression during adhesion clutch and release upon unclutching could be an important factor inducing myosin-II-independent flow fluctuation.

The actin cytoskeleton is a dynamic structure known for its viscoelastic properties, as characterized by its ability to store and dissipate energy—a feature that is central to numerous cellular processes^75^. In our model, viscoelasticity is captured by the incorporation of a damper component, representing the viscous behavior of the F-actin network. To maintain a focused analysis on how elasticity contributes to mechanosensitivity, we have chosen to keep the viscosity parameter relatively not varying. This deliberate simplification allows us to isolate and examine the elastic aspect’s role in cellular responses to mechanical stimuli. The viscous properties of the F-actin network arise from several factors, including the transient binding and unbinding of actin-crosslinking proteins, the polymerization and depolymerization dynamics of actin filaments, and the entropic elasticity of the F-actin meshwork itself. Recent studies have emphasized the importance of viscoelasticity in cellular mechanosensing, suggesting that cells can discern and react to the viscoelasticity of their extracellular matrix (ECM), affecting cell behavior and fate^107,108^. The ability of cells to detect and respond to these mechanical cues is a rapidly expanding area of research, shedding light on the complex interplay between cellular function and the mechanical properties of the cellular microenvironment. Looking forward, our model offers a framework that can be expanded to explore how cells sense viscosity changes in their surroundings. Given the increasing evidence of viscoelasticity’s significance in cell function, an extension of our model to include dynamic viscosity parameters presents a promising avenue for deepening our understanding of cellular mechanotransduction and the multifaceted nature of ECM sensing.

Lastly, actin polymerization is highly coupled with the formation of nascent adhesions^109-111^. Thus, it is possible that differential force transmission in response to different stiffness is sensed at the nascent adhesion level^43^. How different early adhesion molecules are involved in this actin-based mechanosensing would be an interesting direction to further investigate.

## Materials and methods

### Cell culture and reagents.

NIH 3T3 fibroblasts stably expressing mRuby-Paxillin, a generous gift from Dr. Mark H. Ginsberg (University of California, San Diego), were cultured in Dulbecco’s Modified Eagle Medium with phenol red, glutamine. 4.5 g L^−1^ D-Glucose, L-Glutamine, and Sodium Pyruvate (DMEM; F-Corning Incorporated, Corning, NY, USA 10-013-CV) supplemented with 10% fetal bovine serum (FBS; Gibco/Invitrogen, Grand Island, NY, USA; 26140079), 1% penicillin-streptomycin (Gibco/Invitrogen, Grand Island, NY, USA; 15140122) and 1% nonessential amino acids (NEAA; Gibco/Invitrogen, Grand Island, NY, USA; 11140050) in 5% CO_2_, 37 °C condition. SNAP-actin-expressing 3T3 fibroblasts^51^, a generous gift from Dr. Martin Schwartz (Yale University, New Haven, CT, USA), were cultured in DMEM (Corning Incorporated, Corning, NY, USA; 10-013-CV) with phenol red, glutamine. 4.5 g L^−1^ D-Glucose, L-Glutamine, and Sodium Pyruvate, 10% FBS (FBS; Gibco/Invitrogen, Grand Island, NY, USA; 26140079), 1% penicillin-streptomycin (Gibco/Invitrogen, Grand Island, NY, USA; 15140122) and 1% Non-essential amino acids (NEAA; Gibco/Invitrogen, Grand Island, NY, USA; 11140050) and 250 μgmL^−1^ Geneticin (G418 sulfate; Gibco/Invitrogen, Grand Island, NY, USA; 10131035) in 5% CO_2_ at 37 °C. To inhibit myosin-II activity, (−)-Blebbistatin (13013) was purchased from Cayman Chemicals (Ann Arbor, MI, USA). Formin activity inhibitor SMIFH2 (340316-62-3) was purchased from Abcam (Cambridge, United Kingdom, USA). Arp2/3 Complex Inhibitor I, CK-666 (442633-00-3) and Arp2/3 Complex Inhibitor I, Inactive Control, CK-689 (170930-46-8) were purchased from Calbiochem/MilliporeSigma (Burlington, MA, USA). To disrupt actin network, Latrunculin A (76343-93-6) was obtained from Cayman chemicals (Ann Arbor, MI, USA).

### Soft substrate preparation and stiffness characterization.

High-refractive index, soft silicone gels (Q-gel, CHT, Richmond, VA, USA) of different stiffness were fabricated as previously described^112^. Briefly, the gel substrates of five different stiffness, i.e., 0.6, 1.3, 2.7, 6 and 12.7 kPa, were made by thorough mixing of Q-gel 920-part A and B at ratio 1:1, 1:1.1, 1:.1.2, 1:1.5 and 1:2 respectively. The elastic modulus was measured by measuring the shear storage moduli (G’) of the gel at each mixing ratio using a DHR-2 hybrid rheometer (TA Instruments, New Castle, DE, USA). As the Q-gel was very soft and sticky, thus hard to handle, we started to measure the gel’s storage modulus from its pre-cured state on the 40 mm stainless steel Peltier parallel plate stage by a time-sweep test for 10 hours with 0.63 rad s^−1^ oscillation amplitude. The gel was cured to its final stiffness during the test. The metal parallel plate was heated to 80 °C to match the curing temperature of the gels, the gap height was set at 20 μm to accommodate an initially less viscous gel, and strain was set to 1% to stay within the linear viscoelastic regime. This testing was performed via a time sweep program in TRIOS software.

For TFM substrate fabrication, 300 μl of the Q-gel920 A/B mixture was spin-coated on a 35-mm glass-bottom dish with a No. 1.0, 14 mm-diameter circular cover glass (MatTek, Ashland, MA, USA) at 1000 RPM for 30 sec min, followed by curing at 80 °C for 2 hrs. A slower spinning speed, i.e., than the original method (3000 RPM) in^112^, was chosen to increase the gel thickness and to avoid the cells from sensing the rigidness of the glass bottom. The gel thickness was measured by profilometer to be ~45 μm in average. To functionalize the gel surface, (3-aminopropyl) triethoxysilane (APTES, Sigma-Aldrich, St. Louis, MO, USA; 440140) was treated on the coated gel. As fiducial markers for gel deformation visualization, 40-nm carboxylated far-red fluorescent beads (Invitrogen/Thermo Fisher scientific, Waltham, MA, USA; F8789) with a density of 1 bead μm^−2^ (excitation/emission 660/680 nm, Invitrogen, Waltham, MA) were covalently bonded on the gel surface using 1-Ethyl-3-(3-dimethylaminopropyl) carbodiimide (EDC, Sigma-Aldrich, St. Louis, MO, USA; 39391).

### Traction microscopy imaging.

For traction force microscopy experiments, the silicone gel on a glass-bottom dish (MatTek, Ashland, MA, USA) was coated with 10 μg ml^−1^ fibronectin for 30 min at room temperature. 3T3 fibroblasts expressing mRuby-Paxillin were plated on the fibronectin-coated gel substrates. Four hrs after seeding, the beads and the paxillin were imaged under total internal reflection fluorescence (TIRF) microscope (opto-TIRF, CAIRN Research, Faversham ME13 8UP, UK) housed in Nikon Ti-S microscope (Nikon Instruments, Melville, NY, USA) at a 60x TIRF objective. The microscope stage was equipped with an H301 stage-top incubator chamber and UNO controller (Okolab USA Inc, San Bruno, CA, USA) to maintain cells at 5% CO_2_ and 37 °C in a humid environment. The single-shot live cell imaging was performed in phenol-red-free DMEM (Gibco/Invitrogen, Grand Island, NY, USA; 31053028) supplemented with 10% fetal bovine serum (FBS; Gibco/Invitrogen, Grand Island, NY; 26140079), 1% penicillin-streptomycin (Gibco/Invitrogen, Grand Island, NY, USA; 15140122) and 1% nonessential amino acids (NEAA; Gibco/Invitrogen, Grand Island, NY, USA; 11140050). The cells were kept in focus using CRISP autofocus unit (ASI Applied Scientific Instrumentation, Eugene, OR, USA). The far-red fluorescent beads signal and mRuby paxillin signal were imaged at the same focal plane, i.e., on top of the gel, with 642 nm and 587 nm lasers, respectively. The images were captured with a Hamamatsu ORCA-flash 4.0 LT plus sCMOS camera (Hamamatsu Corporation, Bridgewater. NJ, USA) and controlled with MetaMorph imaging software (Molecular Devices, Downington, PA, USA). The bead images with relaxed gel were obtained after removing the cells using 0.5 ml of 10% bleach.

### Perturbations using small-molecular inhibitors.

For TFM experiments, for myosin-II contractility inhibition, 20 μM of blebbistatin (BBS) was applied to cells for 1 hr after 3 hrs of cell seeding on the gel. The BBS concentration and sequence of cell seeding, and treatment were chosen to inhibit most myosin-II ATPase activity with minimum toxicity while ensuring cell adhesion. We found the 20 μM blebbistatin concentration to be optimal for the stiffness-dependent differential traction studies by the following reasons. First, this concentration has been sufficient to inhibit nearly whole myosin contractility. In response to increasing BBS concentrations, the traction force, measured by micropillars, has been found to decrease until 20 μM, after which the average force has become virtually constant with the further increase in the BBS concentration to 40 μM^48^. Second, while in-vitro ATPase activity measurement using spectrophotometer has shown about 80-90% down-regulation of the activity of platelet or non-muscle myosin IIA and IIB activity^113^ in the presence of 20 μM blebbistatin, the concentration higher than 20 μM has shown to be cytotoxic as well as phototoxic on exposure to blue light^50^. Finally, our data indicates that the traction of the BBS-treated cells was further reduced, by 4- or 5-fold, in response to additional inhibition with CK666 or (SMIFH2 or LatA), respectively (Fig. 2). which suggests that the stiffness-dependent differential traction (Fig. 1c) is minimally from remaining active myosin-II activity but mainly from actin polymerization-based actin retrograde flow. Therefore, to inhibit almost all the myosin ATPase activity to in addition to avoiding cytotoxicity to cells, 20 μM concentration of BBS was used.

For inhibition of Arp2/3 complex, 100 μM CK666 was applied to cells in addition to BBS for 1 hr after 3 hrs of cell seeding on the gel. CK666, an Arp2/3 inhibitor used in our study, stabilizes the inactive Arp2/3 complex and block the movement of Arp2 and Arp3 subunits into the active conformation^114^. In our study, we have used CK666 at 100 μM in addition to 20 μM blebbistatin. 100 μM of CK666 has been widely used to effectively down-regulate Arp2/3 activity, which has helped to show reduction in actin density at the leading edge, retraction of lamellipodial actin filament organization, reduction in dense elongated filaments^82^, reduction in actin expression^115^ and in actin polymerization^114^. As a negative control of CK666 treatment, 100 μM of CK689, inactive structural analogue, was applied to cells for an imaging experiment.

To inhibit formin FH2 domain activity, 20 μM SMIFH2 were applied to cells, in addition to BBS, at 3 hours after cell seeding on the gel for 1 hour. SMIFH2 is a small molecule inhibitor that inhibits the nucleation activity of formin homology domain 2. At 20 μM concentration, which we used for the study, SMIFH2 has shown to reduce F-actin bundles in NIH 3T3 fibroblasts^116^. High concentrations of SMIFH2 have exhibited cytotoxicity in different cell lines^117^ including NIH 3T3 fibroblasts^116^. According to Isogai et al. ^117^, administering SMIFH2 at moderate concentrations (<10 μM) and for short treatments (<1 hour) minimizes cytotoxicity. At concentration higher than that, e.g., ~50 μM, SMIFH2 has demonstrated a side-effect to inhibit human non-muscle myosin ATPase activity as well^59^. However, this side-effect does not harm our findings and associated implication because we used less SMIFH2 concentration (than 50 μM) and we anyway applied BBS inhibit myosin-II activity along with formin activity.

Inhibition of actin polymerization itself was done by applying 1 μM latrunculin-A (LatA) along with 20 μM BBS to cells at 3 hours after cell seeding on the gel for 1 hour. LatA binds to actin monomers and prevents them from polymerizing into filaments^118,119^. LatA exposure to NIH 3T3 mouse fibroblasts cells at ~0.9 μM concentration for 1 hour disrupts actin organization^119^. We used 1 μM concentration of LatA to sequester actin monomer and drop down the actin polymerization rate. Treatment of fibroblasts with LatA concentration as low as 0.1 μM has shown disintegration of actin assembly and overall decrease in cell elasticity^120^. LatA has also demonstrated to be more potent at lower concentration (~0.5 μM) and longer times compared to other actin-polymerization inhibitors, Latrunculin-B and cytochalasin D^121^.

The same concentrations of inhibitors were used for the actin speckle imaging experiments. We began imaging 1 hour postcell plating to minimize the presence of well-developed actin fibers, which could interfere with the assessment of lamellipodial actin dynamics. The inhibitor, LatA, was added at the 30-minute mark after plating. Following a 30-minute period postinhibitor treatment, imaging was conducted to capture the actin flow.

### Traction reconstruction.

From a pair of bead images acquired in the presence and absence of cells, traction was reconstructed using our MATLAB-based TFMPackage software^44,45^. Briefly, the displacement field was calculated by a cross-correlation-based particle tracking velocimetry with retracking (cPTVR) method that is able to track large, local displacement^42^. The force reconstruction was performed using Fast Boundary Element Method (FastBEM) with L_2_-norm-based regularization where a regularization parameter was chosen based on L-curve, L-corner method. Acquired traction fields were interpolated over the original microscopic image area and quantified for an average traction over a 2 μm-thick perimeter band area from a cell segmentation captured from corresponding mRuby-paxillin channel images.

### Actin fluorescence speckle imaging.

For time-lapse live-cell imaging of actin speckles, SNAP actin-expressing 3T3 fibroblasts were labeled by culture in a 24-well culture dish up to 70% confluency followed by incubation with 0.5 μM SNAP-Cell 647-SiR (New England Biolabs, Ipswich, MA, USA; S9102S) at 37 °C for 30 min. Cells were then washed thoroughly with the DMEM phenol-red-free media every 30 min for 2 hrs. They were incubated at 37 °C for 30 min after every wash. SNAP-Cell 647-SiR labelled cells were then seeded on fibronectin-coated silicone gel on top of glass-bottom dishes (#1, 14-mm-diameter glass coverslip, 35-mm dish: MatTek, Ashland, MA, USA). Actin-speckle imaging was performed after 45 min-1 hr of cell seeding. Actin speckles were imaged under a spinning-disk confocal microscopy, a Nikon Ti-S microscope equipped with Yokogawa spinning disk head (CSUX1), stage-top based incubation chamber system (OkoLab, Ambridge, PA, USA), XY motorized stage with linear encoders (ASI Applied Scientific Instrumentation, Eugene, OR, USA), a focus-drift-compensation system (CRISP with 780 nm LED), and a high-resolution, high-frame-rate camera (ORCA-Flash LT sCMOS). A laser line with 642 nm wavelength was used for exciting SNAP-actin-SiR647, and cells were imaged under a 100x objective for 3 mins with a time interval of 6 seconds (64.5 nm per pixel, NA = 1.4, 16-bit images).

### Actin retrograde flow quantification from speckle images.

Quantification of time-lapse actin speckle movies was performed using quantitative fluorescence speckle microscopy (qFSM) software in MATLAB (MathWorks)^52,122^. First, images acquired at 6 frames per minute were calibrated using noise model calibration. Cell masks were generated using manual thresholding. Speckles were detected by setting the alpha value for statistical selection of speckles (0.05) with maximum iteration at 3. Flow tracking was performed on 1 to 31 frames with a 2-frame integration window, 1-frame step size, template size range 17-35 pixels, maximum flow speed 10 pixels frame^−1^. As additional settings, mask edge erosion width was set at 5 pixels, and the relative distance for filtering vector outlier in respect to local neighborhood was set to 1. Speckles were tracked by performing a hierarchical tracking using nearest neighbor flow with search radius of 3 pixels and a correlation length of 33 pixels. Flow analysis was performed using speckle tracking as the flow process to analyze by time averaging for 3 number of frames, correlation length of 33 pixels and grid size width of 11 pixels. For SMIFH2 and latrunculin-A actin-speckles time-lapse images, PIV was used for flow quantification instead of particle tracking because not many individual speckles were detectable using Gaussian fit-based detection. A potential stage drift was checked for each movie by a user and by a software. Actin flow speed was quantified in the five different layers of the cell from the cell edge with 1μm in thickness for each layer.

### Atomic force microscopy (AFM) force spectroscopy.

Adherent 3T3 fibroblasts were seeded on 12.7 kPa silicon gels coated with 5 μgmL^−1^ of fibronectin for a total of 4 hours (with and without relevant small molecule pharmacological perturbations). Atomic Force Microscopy (AFM) force spectroscopy experiments were performed using a Bruker BioScope Resolve AFM system mounted on an inverted Axiovert 200 M microscope (Zeiss) equipped with a 40x objective lens (0.6 NA, LCPlanFl, Olympus). The microscope system is on an acoustic isolation table. During AFM experiments, cells were maintained at 37 °C using a heated stage (Bruker). A modified AFM microcantilever attached with a 10-μm-diameter polystyrene bead (Novascan) was used for all AFM measurements. All AFM microcantilevers were pre-calibrated using the standard thermal noise fluctuations calibration method. The calibrated spring constant was 0.095 Nm^−1^ - 0.1 N m^−1^. For lamellipodium measurements, up to five force curves were performed in succession with a 10 sec delay between each measurement. Depending on the cell and treatment condition, the applied force was set to be between approximately 400 pN - 1.2 nN, typically yielding indentations between 100 and 200nm. The force curves ramp rate was set to 1 Hz yielding AFM probe approach/compressive speeds between 1.5 μm s^−1^ - 2 μm s^−1^. The cell’s lamellipodium Young’s modulus (Pa) were analyzed and determined using the Bruker NanoScope Analysis software. In brief, force curves were corrected for the non-contact region slope (typically arising from the hydrodynamic drag and AFM probe-sample orientation) using a baseline subtraction function. Then, we used the Hertz contact mechanics methods for rigid spherical prove indenting an infinite isotropic elastic half-space to calculate the Youngs’ modulus^123^. Only the first 90% of the approach curve was considered for the analysis.

### Statistical analysis.

Comparison of normalized traction (Fig. 2g) and normalized flow speed (Fig. 3g) between blebbistatin (BBS) and CK666-BBS treated cells was done using Mann-Whitney U nonparametric test because most of the data were non-Gaussian when tested by Kolmogorov-Smirnov test. Comparison of normalized traction (Fig. 2h) and normalized flow speed (Fig. 3h) among CK666-BBS, SMIFH2-BBS and LatA-BBS treated cells was done using Kruskal-Wallis ANOVA and Dunn’s post-hoc analysis.

### A traditional molecular clutch model assuming rigid actin treadmilling with addition of actin polymerization as another source of traction

#### Model parameters and implementation.

To analyze the force transmission and actin velocity in response to different ECM stiffness, the original molecular clutch model was first developed by Chan et al. ^22^, which was modified further by including an adhesion reinforcement^18^. Our model is based on this model, which we further modified with additional component of actin’s own force-velocity relationship. We refer this modified model^18^ as the traditional model for description purpose.

#### Common parameters shared in the traditional model.

The traditional model considers a given number of myosin motors ($n_{m}$) pulling on the F-actin filament generating the characteristic retrograde flow of actin towards the cell center by exerting a force F_m_. The F-actin filament can bind to a given number of fibronectin (FN) molecules ($n_{f}$) on the substrate through talin-integrin clutches, which can be modelled as linear elastic hooks in parallel with a spring constant $k_{c}$ (Fig. 4a). Fibronectin’s own elasticity was ignored, i.e., regarded rigidly connected to the elastic substrate with spring constant $k_{\text{sub}}$. In the model, FN molecules are allowed to reversibly associate to the integrins with an effective binding rate $k_{on}$ and disassociate according to an effective unbinding rate $k_{off}$. As the number of FN molecules are considered fixed in the model, the effective binding rate is given by $k_{on}=k_{ont}\cdot d_{\text{int}}$ where $k_{\text{ont}}$ is the true binding rate characterizing integrin-fibronectin interaction, and $d_{\text{int}}$ is the density of integrins on the cell membrane.

The simulation begins with unbound clutch-FN linkages, i.e., the absence of adhesion to the substrate, with unloaded myosin motors. In this model, due to myosin’s force-velocity relationship,

(1) $$V_{u}=V_{a}\left(1-\frac{F_{\text{sub}}}{n_{m}F_{m}}\right),$$

where $F_{m}$ is the force required to stall a myosin motor and thus $n_{m}\cdot F_{m}$ is the maximum isometric tension by myosin motors and $F_{\text{sub}}$ is the traction on the substrate, which is equivalent to the tension on the myosin due to force balance, F-actin filaments flow with the maximum rearward speed $V_{a}$. Then, in the subsequent time step, the number of bound clutches grows because $k_{\text{off}}$ rate is not sufficient to exceed $k_{\text{on}}$ rate due to insufficient tension in the myosin. The bound clutches are pulled by the rearward movement of F-actin filaments, $V_{u}$, thus exerting force to the substrate. After every time step, the total traction force applied to the substrate $F_{\text{sub}}$ is calculated by applying force balance:

(2) $$F_{\text{sub}}=\frac{k_{\text{sub}}k_{c}\sum_{i=1}^{n_{\text{bound}}}x_{i}}{k_{\text{sub}}+n_{\text{bound}}k_{c}},$$

where $x_{i}$ is the position of each bound molecule, and $n_{\text{bound}}$ is the total number of bound molecules. The retrograde speed $V_{u}$ is recalculated by the updated $F_{\text{sub}}$ and the linear force-velocity relationship (Eq. 1), i.e., the F-actin speed slows down due to the increased force.

To compare model predictions with experiments, the force applied to the substrate $F_{\text{sub}}$ and spring constant $k_{\text{sub}}$ were converted to cell traction stress $P_{\text{sub}}$ and Young’s modulus E, respectively, by assuming a given adhesion radius $r_{a}$. The equations used are:

(3) $$E=\frac{9k_{\text{sub}}}{4\pi r_{a}},$$

(4) $$P_{\text{sub}}=\frac{F_{\text{sub}}}{\pi a^{2}},$$

which are the same as used in Elosegui-Artola et al. ^18^. The area of the adhesion (${\pi r}_{a}^{\;\;2}$) represents thus the total surface occupied by the number of fibronectin molecules $n_{f}$ considered in the simulation.

For myosin-independent force transmission simulation, we added the actin polymerization’s contribution to the retrograde flow by adding a new parameter, $v_{\text{actin}}$,

(5) $$v_{f}=v_{u}*\left(1-\frac{k_{\text{sub}}*x_{\text{sub}}}{F_{\text{s,myosin}}}\right)+v_{\text{actin}}*\left(1-\frac{k_{\text{sub}}*x_{\text{sub}}}{F_{\text{s,actin}}}\right),$$

where $v_{actin}$ is the unloaded actin-polymerization-driven actin flow speed and $F_{\text{stall},\text{actin}}$ is the maximum force required to stall the actin flow by $n_{af}$ F-actin filaments, thus $F_{\text{stall},\text{actin}}=n_{\text{af}}F_{\max}$, where

(6) $$F_{\max}=\frac{k_{B}T}{\delta }\;\text{ln}\left(\frac{C}{C_{crit}}\right)$$

is a stall force by a single F-actin filament and is assumed to be equivalent to the stall force determined by the Brownian ratchet model^124^. The expression contains $k_{B}$ as Boltzmann’s constant, T as the absolute temperature, δ as the elongation distance for addition of a single protein subunit and is 2.7 nm for actin, C as the concentration of monomers in solution, and $C_{\text{crit}}$ as the critical concentration for polymerization. For $C_{\text{crit}}$, 0.12 μM was used as it was measured for pure ATP-actin in standard polymerization buffer at the barbed end of a filament^125^. For C, we use the total typical concentration of actin, 100 μM^126^. Vu was kept zero, reflecting no myosin contraction.

In our model with actin polymerization, the clutch unbinding rate, $k_{\text{off}}$, was adjusted to recapitulate high flow speed in CK666-BBS condition by increasing the unclutching rate magnitude overall to 6 compared to 0.5 in BBS (Fig. 4e, f.). $k_{\text{off}}$ was modeled with an exponential corresponding to the experimental data from Kong et al. ^127^.

(7) $$k_{\text{off}}=a\;\text{exp}\left(bF_{c}\right),$$

where the constants a and b were adjusted depending on the range of $F_{c}$, *i.e.*, a=0.1905 and $b=2.333\times 10^{11}$ if $F_{c}<13.2\times 10^{-12}\;N$, and a=0.04527 and $b=7.251\times 10^{10}$ if $F_{c}<30\times 10^{-12}\;N$.

The model includes the rate constant functions for talin unfolding ($k_{\text{uf}}=\text{exp}(-7.573)\cdot \text{exp}(1.786\times 10^{12}\;F_{c}p_{t})$), refolding ($k_{f}=\text{exp}(13.07)\cdot \text{exp}(-2.288\times 10^{12}\;F_{c}p_{t})$), and as a function of the force in the clutch ($F_{c}$) and the fraction of clutch force experienced by talin ($p_{t}=0.073$). The model also includes a binding rate of vinculin to talin ($k_{\text{onv}}=10^{8}$) These were held consistent with the values used in the previous clutch model^21^.

### Actin-elasticity-based molecular clutch model.

In this modified model, the F-actin network unit was modeled as a mesoscale viscoelastic material with a length L with an individual spring constant of $k_{\text{actin}}$ and viscosity of η. Rather than being controlled by a constant velocity determined by the force-velocity relationship (Eq. 5), the flow in this model is driven by the force equilibrium between the F-actin filament, the clutches, and the substrate. The force generated in the purely elastic spring portion of the actin is generated by the force balance with the elastic clutch, and is found to be:

(8) $$k_{\text{actin}-k}=\frac{N_{n}L-X_{c}}{N_{o}+N_{n}}k_{\text{actin}},$$

where $N_{n}$ is the number of new actin units added in that time step, and $N_{0}$ the number of units in the original filament at the start of the first time-step. $N_{n}$ is modeled to be inversely related to the maximum force in any of the individual clutches denoted as $F_{c}$:

(9) $$N_{n}=N_{n,\max}\left(1-\frac{F_{c}}{F_{s,\text{actin}}}\right)$$

where $F_{s,\text{actin}}$ is the stall force of actin addition, and $N_{n,\max}$ is the maximum number of actin units that can be added in a single time step. The force in the substrate and clutch are described by following equations:

(10) $$F_{\text{sub}}=k_{s}*x_{\text{sub}}$$

(11) $$F_{\text{adh}}=N_{c}K_{c}(x_{c}-x_{\text{sub}})$$

where $F_{\text{adh}}$ is the force in the clutch, and $N_{c}$ is the number of bound clutches. As the clutches are attached to the substrate, the force balance between the clutches and the substrate can be used to create the expression for the displacement of the substrate:

(12) $$X_{\text{sub}}=\frac{N_{c}k_{c}X_{c}}{k_{s}+k_{c}N_{c}}.$$

The rate of change of the position of the clutch is derived using following equations:

(13) $$\eta {\dot{x}}_{c}=F_{\text{actin}-k}-F_{\text{adh}}$$

(14) $${\dot{x}}_{c}=\frac{1}{\eta }\left(\frac{N_{n}L-X_{c}}{N_{o}+N_{n}}.k_{\text{actin}}-N_{c}k_{c}(X_{c}-X_{\text{sub}})\right).$$

The time derivative of the clutch position is explicitly integrated in the model using the Euler method to find the displacement following each time step of the simulation. The maximum rate of displacement of any of the clutches is considered to be the actin rearward speed. The displacement is used to calculate the force exerted on the substrate using Eqs. (13) and (10), which is used to find the traction exerted on the substrate using Eq. (3). Main parameters, their values per condition and references are summarized in Supplementary Table 3.

## Supplementary Material

Supplementary Information

Supplementary Movie 1

Supplementary Movie 2

Supplementary Movie 3

Supplementary Movie 4

Supplementary Movie 5

## Data Availability

The data and set of MATLAB codes and functions used for the modeling are available via our GitHub site (https://github.com/HanLab-BME-MTU/actinElasticClutchModel.git). The raw images and processed data for TFM and qFSM experiments are shared via a repository in Open Science (https://osf.io/pt53b/?view_only=d216fb0d804a4806a3ef81eaf96a7bc0).
