# Supplementary material for: Myosin-independent stiffness sensing by fibroblasts is regulated by the viscoelasticity of flowing actin
Source: Commun Mater. Author manuscript; Available in PMC 2024 May 13. (PMC11090405; doi:10.1038/s43246-024-00444-0)
Supplement: Supplementary Information [file NIHMS1988538-supplement-Supplementary_Information.pdf]

Supplementary Information

Myosin-independent stiffness sensing by fibroblasts is regualted by the viscoelasticity of flowing actin

Short title: Myosin-independent, stiffness-dependent traction

Nikhil Mittal[1,5], Etienne B. Michels[1], Andrew E. Massey[2], Yunxiu Qiu[3], Shaina Royer-Weeden[1], Bryan R. Smith[3], Alexander X. Cartagena-Rivera[2], Sangyoon J. Han[1,4,5]

List:

- Supplementary Figure 1
- Supplementary Figure 2
- Supplementary Figure 3
- Supplementary Figure 4
- Supplementary Figure 5
- Supplementary Figure 6
- Supplementary Table 1
- Supplementary Table 2
- Supplementary Table 3
- Supplementary References

Supplementary Figure 1

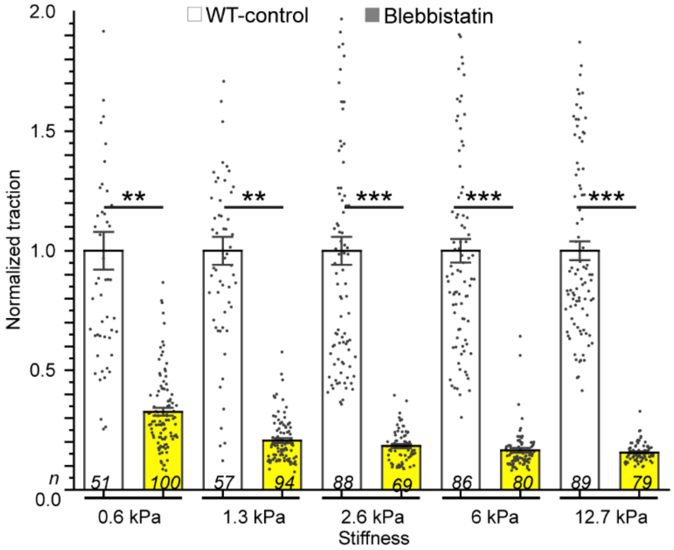

Supplementary Figure 1. Comparison of normalized traction between WT NIH 3T3 fibroblasts (white) and BBS-treated cells (yellow) treated fibroblasts per different gel stiffness. Bar with error bar: mean ± SD. **: $p<0.05$, **: $p<1\times10^{-30}$, ***: $p<1\times10^{-50}$* by Mann-Whitney U test. Sample sizes, *n,* are denoted on bottom of each bar. Markers with error bars: mean ± s.e.m.

Supplementary Figure 2

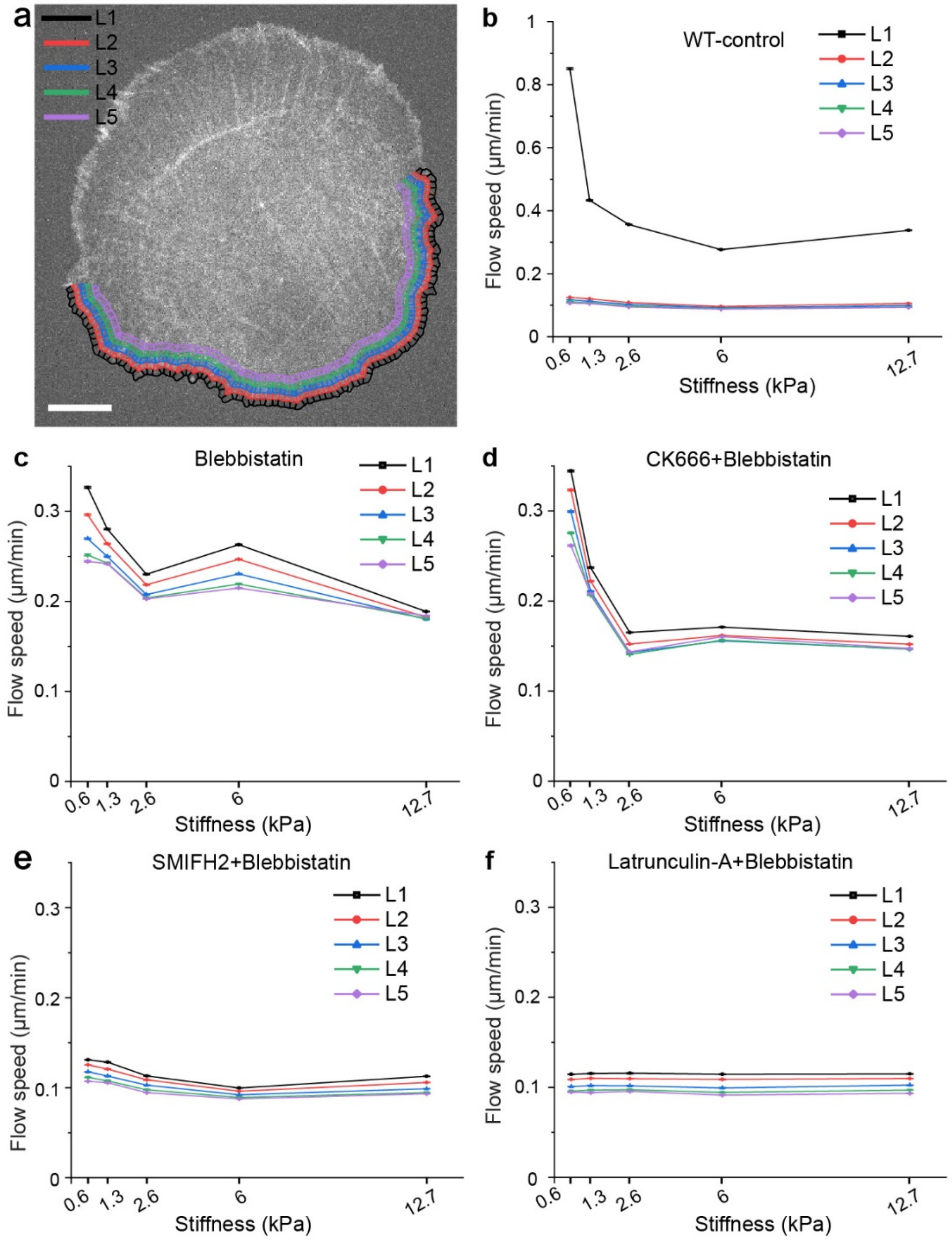

Supplementary Figure 2. Five 1 µm-thick layers from the cell edge containing many windows to sample actin flow speed and associated speeds per layer per drug condition. (a) A representative image of SNAP-actin-SiR647 of a WT fibroblast overlaid with windows of the five color-coded layers. Scale bar: 10 µm. (b-f) Average F-actin flow speeds as a function of the gel stiffness in the five layers of WT-control cells (b, n = 7, 7, 7, 13, 13 cells for increasing stiffness, collected from m = 44879, 51583, 43368, 80533, 53718 windows), cells treated with 20 µM BBS (c, n = 7, 9, 9, 9, 9 cells for increasing stiffness, m = 50798, 66822, 65920, 62777, 57369 windows), cells treated with 100 µM CK666 in addition to 20 µM BBS (d, *n* = 9, 10, 10, 9, 9 cells for increasing stiffness, collected from *m* = 67937, 75571, 69670, 85238, 65224 windows), cells treated with 20 µM SMIFH2 in addition to 20 µM BBS (e, *n* = 8, 11, 12, 14, 9 cells for increasing stiffness, collected from *m* = 57937, 42861, 79091, 99196, 80533, 54022 windows), and cells treated with 1 µM LatA in addition to 20 µM BBS (f, *n* = 9, 10, 8, 8, 12 cells for increasing stiffness, collected from *m* = 52341, 52948, 36332, 45756, 65410 windows). Markers with error bars: mean ± s.e.m.

Supplementary Figure 3

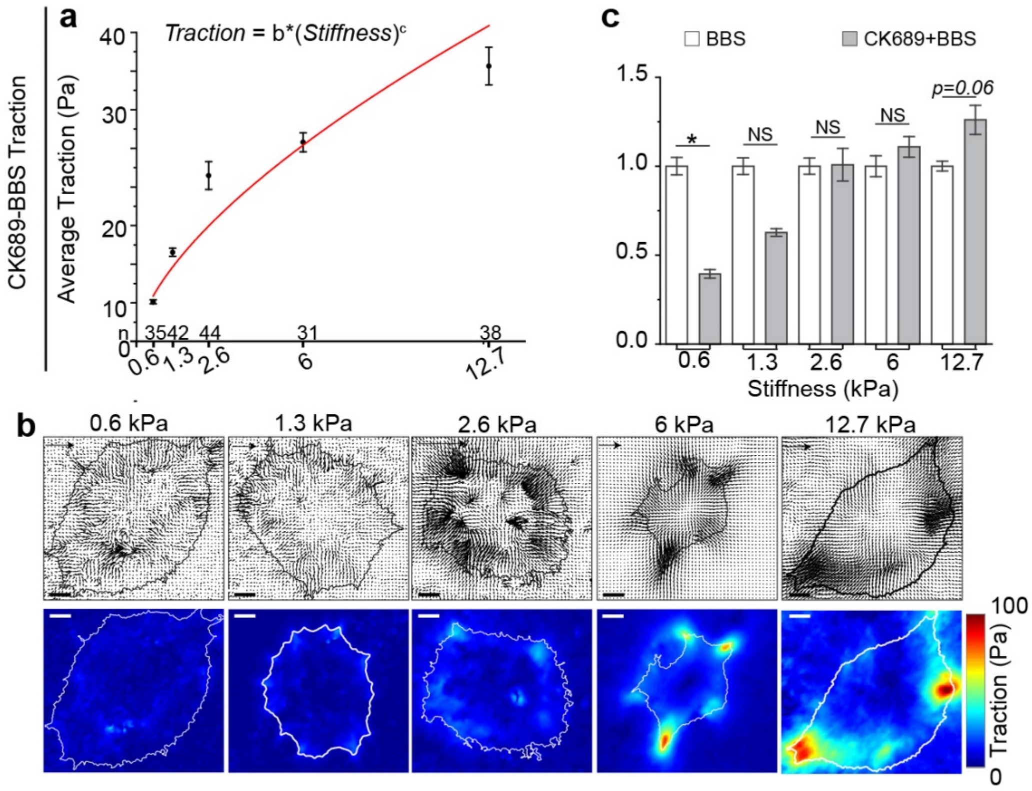

Supplementary Figure 3. Stiffness-dependent differential force transmission is not affected by the inactive inhibitor CK689. (a) Average traction integrated over 2-µm-thick cell perimeter of CK689-BBS treated fibroblasts as a function of different gel stiffness (red). Sample sizes, *n,* are denoted on top of each stiffness value. Markers with error bars: mean ± s.e.m. **(b)** Representative traction vector fields (top) and traction magnitude maps (bottom) of WT-control cells. Arrow scale: 50 Pa, 75 Pa, 90 Pa, 100 Pa and 150 Pa of traction for gel stiffness of 0.6 kPa, 1.3 kPa, 2.6 kPa, 6 kPa and 12.7 kPa, respectively. Scale bar: 10 µm. Power-law curve fits (*Traction* = b*(*Stiffness*)^c) was added in **(a)** (See Supplementary Table 1 for fit parameters). **(c)** Normalized average traction of CK666-BBS-treated cells (white) and CK689-BBS (grey). Bar with error bars: mean ± s.e.m., *: $p<0.05$ by Mann-Whitney U test.

Supplementary Figure 4

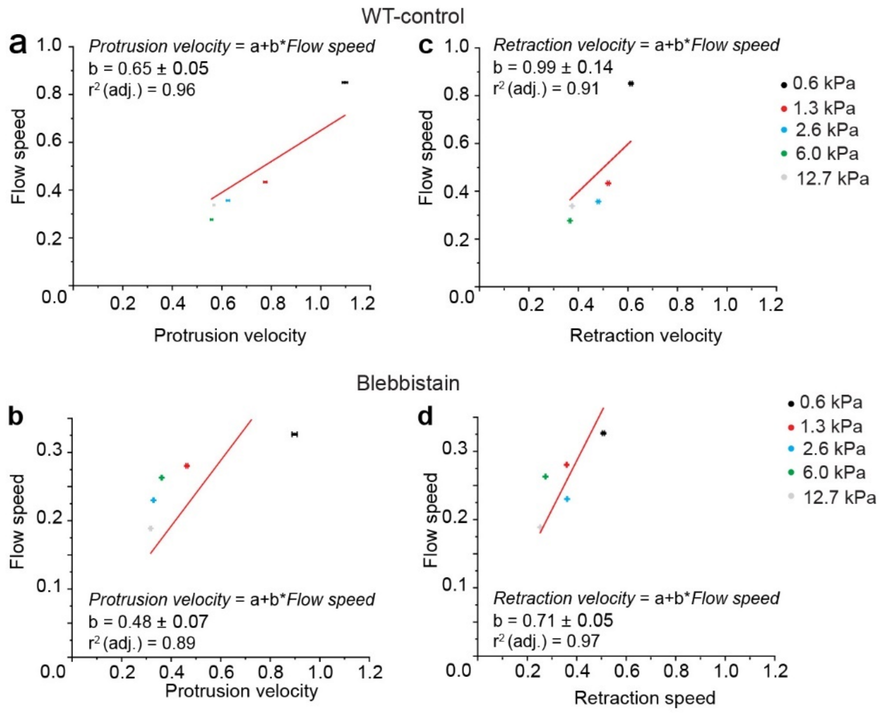

Supplementary Figure 4. Retrograde flow speed is proportional with cell protrusion speed in response to gel stiffness demonstrating balanced contribution of actin polymerization. (a) Average F-actin flow speed as a function of the average protrusion velocity of WT control cells on different gel stiffness (speckles collected from m = 44879, 51583, 43368, 80533, 53718 windows and protrusion speed from *o* = 3507, 3507, 3507, 6513, 6513 windows). **(b)** Average F-actin flow speed as a function of the average retraction velocity of WT control cells on different gel stiffness (retraction speed from *o* = 4907, 4907, 4907, 9113, 9113 windows). Protrusion, retraction, and F-actin flow speed collected from WT cells *n* = 7, 7, 7, 13, 13 cells for 0.6 kPa (*black*), 1.3 kPa (*red*), 2.6 kPa (*blue*), 6 kPa (*green*), and 12.7 kPa (*grey*). **(c)** Average F-actin flow speed as a function of the average protrusion velocity of BBS-treated cells on different gel stiffness (speckles collected from *m* = 50798, 66822, 65920, 62777, 57369 windows and protrusion speed from *o* = 6513, 6513, 6513, 6513, 6513 windows). **(d)** Average F-actin flow speed as a function of the average retraction velocity of BBS-treated cells on different gel stiffness (retraction speed from *o* = 9113, 9113, 9113, 9113, 9113). Protrusion, retraction, and F-actin flow speed collected from BBS-treated cells *n* = 7, 9, 9, 9, 9 cells for 0.6 kPa (*black*), 1.3 kPa (*red*), 2.6 kPa (*blue*), 6 kPa (*green*), and 12.7 kPa (*grey*). Markers with error bars: mean ± s.e.m. Linear fit *flow speed = a + b*protrusion speed* was used to fit flow speed *vs* protrusion speed data, where a= 0 and b= 0.65 ± 0.05 with R^2 = 0.9717 for WT control; a= 0 and b= 0.48 ± 0.07with R^2 = 0.9159 for BBS. Linear fit *flow speed* = a + b**retraction speed* was used to fit flow speed *vs* protrusion speed data, where *a*= 0 and *b*= 0.99 ± 0.14 with R^2 = 0.9276 for WT control; *a*= and *b*= 0.72 ± 0.05 with R^2 = 0.9789 for BBS.

Supplementary Figure 5

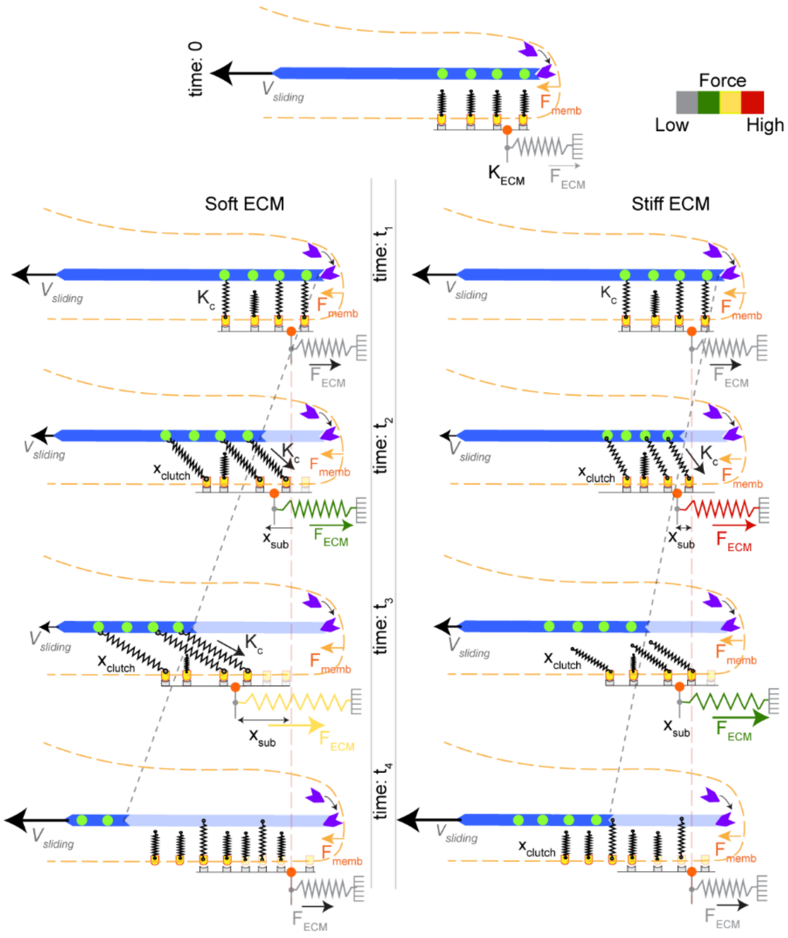

Supplementary Figure 5. Schematic of rigid-actin-based molecular clutch model on soft and stiff substrate. Actin cables are connected to the ECM through several parallel clutches formed by adapter proteins, integrins and fibronectin. Myosin-based force was ignored. Instead, the model considers retrograde flow velocity generated by the actin-polymerization $v_{actin} = v_{max,actin}(1 - F/F_{stall,actin})$. At *t=0*, all the clutches are disengaged, and the actin filaments (*blue)* flows with a rearward speed V_{max}. At $t=t_1$, the clutches begin to engage in both soft and stiff substrate. At $t= t_2$, the engaged clutches begin getting stretched by the retrograde movement of the actin filament *(shown by green points joining clutch and actin flow)*. At $t= t_3$, *on Left: soft substrate*, tension develops slower due to the substate being softer which results in faster actin flow velocity. *Right: stiff substrate,* tension develops faster as substrate is stiffer resulting in smaller actin flow velocity. At $t= t_4$, new cycle of clutching-unclutching begins.

Supplementary Figure 6

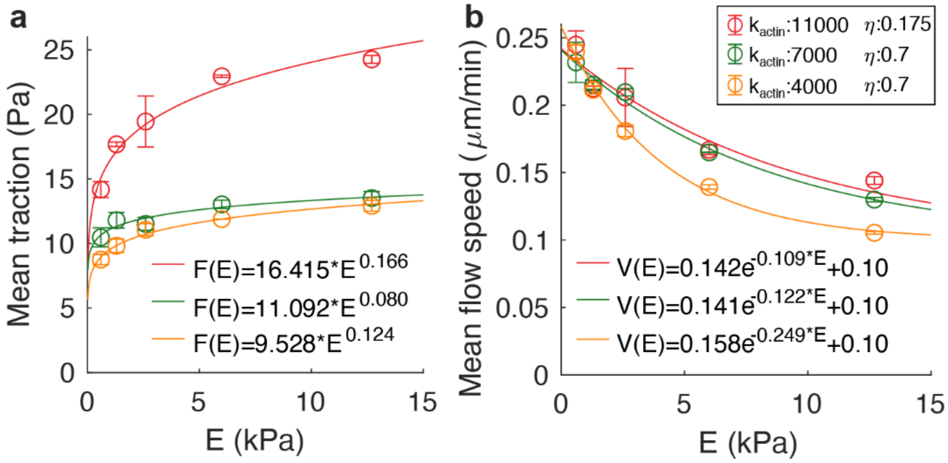

Supplementary Figure 6. Model prediction for traction (a) and retrograde flow speed (b) of BBS- (*red*) and CK666-BBS-treated cells (*green* or *orange*). The difference for CK666-BBS-treated case is that k_{actin} = 7000 (*green*) or 4000 (*orange*) were used as compared to the value (k_{actin} = 1500) used in the main manuscript, *i.e.*, Fig. 5e,f. The viscosity η needed to be increased to accommodate less flow speed by dampening.

Supplementary Tables

Supplementary Table 1. Nonlinear regression coefficients of the average force of 3T3s with respect to substrate shown in control, presence of myosin-inhibitor Fig. 1 a & c and in presence of Arp2/3-myosin, formin-myosin, and polymerization-myosin inhibitor in Fig. 2 a, c, e respectively. Fit function for model: Traction = **b***(Stiffness)$^{\mathbf{c}}$

Parameters / Conditions	b	c	Adjusted R^2
**WT-control**	52.82 ± 6.41	0.51±0.07	0.93
**Blebbistatin**	12.67 ± 0.46	0.34±0.02	0.99
**CK666-Blebbistatin**	2.30 ± 0.16	0.47±0.05	0.96
**SMIFH2-Blebbistatin**	1.24 ± 0.02	0.52±0.02	0.99
**Latrunculin A-Blebbistatin**	1.16 ± 0.11	0.55±0.05	0.96
**CK689-Blebbistatin**	8.14 ±0.91	0.63 ±0.08	0.90

Supplementary Table 2. Nonlinear regression coefficients of the flow speed of 3T3s with respect to substrate shown in control, presence of myosin-inhibitor Fig. 1 e & f , and in presence of Arp2/3-myosin, formin-myosin, and polymerization-myosin inhibitor in Fig. 3 a, c, e respectively. Fit function for model: $V = \mathbf{a}*\exp(\mathbf{R_0}E) + \mathbf{V_0}$ where $\mathbf{V_0}$ is **initial velocity,** V is final velocity, and E is Young's modulus of the gel.

Parameters / Conditions	V_0	a	R_0	Adjusted R^2
**WT-control**	304.48±24.05	1568.92±1038.26	-1.81±0.79	0.76
**Blebbistatin**	214.49±19.92	270±0	-1.16±0.49	0.46
**CK666-Blebbistatin**	163.49±5.69	454.09±115.53	-1.49±0.34	0.96
**Smifh2-Blebbistatin**	104.89±6.18	41.82±22.89	-0.65±0.65	0.56
**Latrunculin A-Blebbistatin**	114.80±0.74	-0.51±1.29	-0.40±2.75	-1.81

Supplementary Table 3. Main parameters for actin-elasticity based molecular clutch model.

Parameters	Description	Values	Condition	References
n_m	Number of myosin motors	1200	WT-control	Molly et al.[1], Elosegui-Artola et al.[2,3], Chan et al.[4], Oria et al.[5]
		0	BBS, CK666-BBS, SMIFH2-BBS, LatA-BBS	
F_m	Stall force of 1 myosin motor (N)	$-2x10^{12}$	WT-control	
v_u	Unloaded myosin motor velocity (m/s)	$-15\ x10^{-9}$:	WT-control	
		0	BBS, CK666-BBS, SMIFH2-BBS, LatA-BBS	
n_c	Number of molecular clutches (fibronectin molecules)	1200	All conditions	
d_{int}	Density of integrin molecules (#/μm^2)	300<br>60	WT-control<br>BBS	
k_{ont}	On-rate of integrin activation and binding (μm^2/s)	2.83×10^{-4}<br>2.11×10^{-4}	WT-control<br>BBS	Elosegui-Artola et al.[2,3], Oria et al.[5]
k_{off}	Unbinding rate of integrin (s^{-1})	0.2<br>0.8	WT-control<br>BBS	Elosegui-Artola et al.[2,3], Oria et al.[5], Kong et al.[6]
k_{onv}	Binding rate between vinculin and unfolded talin (s^{-1})	10^8	All conditions	Elosegui-Artola et al. [3]
k_c	Clutch spring constant (N/m)	1 N/m	All conditions	Elosegui-Artola et al.[3], Oria et al.[5], Chan et al.[4]
int_{add}	Number of integrins added per sq. micron every time reinforcement happens (μm^2/s)	2.4<br>0	WT-control<br>BBS	Elosegui-Artola et al.[2,3]

k_{sub}	Substrate spring constant (N/m)	$[0.1\times10^{-3}$ - $2\times10^{-3}]$	All conditions	Chan et al.[4]
v_{actin}	Unloaded actin-polymerization-driven actin flow speed (nm/s)	-6	WT-control	Yumura et al.[7]
		-6	BBS	
		-1	BBS+CK666, k_{off}=0.5	
		-6	BBS+CK666, k_{off}=3	
n_{af}	The number of actin filaments (#/segment)	56.2<br>56.2<br>7.2	WT-control<br>BBS<br>BBS+CK666	Introduced (Used only in the traditional clutch model)
$F_{s,actin}$	force required to stall actin flow (pN)	567<br>567<br>73	WT-control<br>BBS<br>BBS+CK666	Introduced (Used only in the traditional clutch model)
k_{actin}	Spring constant of individual actin unit (nN/mm)	$1 \times n_{af}$	All conditions	Gardel et al.[8] (Used only in the actin elasticity-based clutch model)
L	Length of the individual actin unit (nm)	32	All myosin-free conditions	Introduced (Used only in the actin elasticity-based clutch model)
d	Distance from the edge to the closest integrin adhesion (μm)	1	All myosin-free conditions	Ponti et al.[9]

Supplementary References

1 Molloy, J., Burns, J., Kendrick-Jones, J., Tregear, R. & White, D. Movement and force produced by a single myosin head. *Nature* **378**, 209-212 (1995).

2 Elosegui-Artola, A. *et al.* Rigidity sensing and adaptation through regulation of integrin types. *Nature materials* **13**, 631-637 (2014).

3 Elosegui-Artola, A. *et al.* Mechanical regulation of a molecular clutch defines force transmission and transduction in response to matrix rigidity. *Nature cell biology* **18**, 540-548 (2016).

4 Chan, C. E. & Odde, D. J. Traction dynamics of filopodia on compliant substrates. *Science* **322**, 1687-1691 (2008).

5 Oria, R. *et al.* Force loading explains spatial sensing of ligands by cells. *Nature* **552**, 219-224 (2017).

6 Kong, F., García, A. J., Mould, A. P., Humphries, M. J. & Zhu, C. Demonstration of catch bonds between an integrin and its ligand. *Journal of Cell Biology* **185**, 1275-1284 (2009).

7 Yumura, S. Myosin II dynamics and cortical flow during contractile ring formation in Dictyostelium cells. *The Journal of Cell Biology* **154**, 137-146 (2001).

8 Gardel, M. L. *et al.* Elastic behavior of cross-linked and bundled actin networks. *Science* **304**, 1301-1305 (2004).

9 Ponti, A., Machacek, M., Gupton, S., Waterman-Storer, C. & Danuser, G. Two distinct actin networks drive the protrusion of migrating cells. *Science* **305**, 1782-1786 (2004).
